# Three-Dimensional Response-Surface Models to Predict the Diameter of Electrospun Polycaprolactone Nanofibers: Application in the release of ferulic acid

**DOI:** 10.1021/acsomega.6c03403

**Published:** 2026-07-24

**Authors:** Felipe Lestón-Cabeo, Nuria Muñoz-Flores, Melissa Olmedo-Navarro, Francisco M. Arrabal-Campos, Ignacio Fernández, Rafael Contreras-Cáceres

**Affiliations:** Department of Chemistry and Physics, Research Centre CIAIMBITAL, 396776 University of Almería, Almería 04120, Spain

## Abstract

Controlling the diameter of electrospun polymeric nanofibers is essential for optimizing their performance in localized drug delivery applications. In this context, mathematical models able to use processing parameters to predict nanofiber dimensions remain limited. In this work, a series of polycaprolactone nanofibers synthesized across a wide diameter range (727–2925 nm) were initially fabricated by the electrospinning method to be subsequently evaluated for localized drug release applications. The nanofiber diameter was systematically varied by adjusting the flow rate and the solvent composition during nanofiber fabrication, while keeping the polymer mass constant. After that, three-dimensional response-surface models were developed, revealing a logarithmic dependence of the nanofiber diameter on the flow rate and an inverse dependence on the solvent ratio. Importantly, both the polymer mass flux through the Taylor cone and the solution viscosity were found to be derived variables, rather than independent, governed by the flow rate and solvent composition, respectively. To connect nanofiber fabrication with drug delivery performance, ferulic acid was selected as a model bioactive compound, and it was incorporated into the nanofibers at three loading levels (0.5, 2, and 6 wt %). Release studies, monitored by high-performance liquid chromatography and ultraviolet–visible spectroscopy, showed that the release behavior was strongly dependent on the ferulic acid content and on the electrospinning processing conditions, whereas no direct dependence on the nanofiber diameter alone was observed. Finally, a kinetic analysis using Higuchi, Peppas–Sahlin, and first-order models revealed that a purely diffusive mechanism does not apply. Instead, the release follows either a first-order or a Peppas–Sahlin model depending on the flow rate used during fabrication. These findings demonstrate that the processing parameters that govern nanofiber diameter also control the internal drug distribution and, consequently, the release kinetics, providing a unified framework for the rational design of electrospun drug delivery systems.

## Graphical abstract

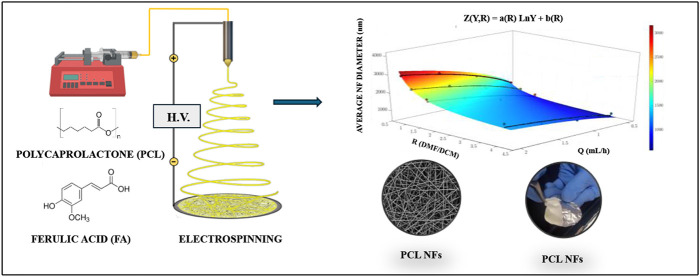

## Introduction

Polymeric nanofibers (NFs) are widely used for numerous biomedical applications, including tissue engineering, regenerative medicine, localized drug release, and cancer therapy.^1,2^ In this sense, electrospinning is the most common technique for the fabrication of polymeric NFs, producing macroscopic structures that are used as dressing materials or polymeric mats in the aforementioned areas.^3,4^ Electrospinning is simple and generates nanofibers having a large surface-to-volume ratio, high porosity, flexible behavior, controllable morphology, and high mechanical capabilities.^5–7^ Briefly, a strong electrostatic field applied to a polymeric solution deforms the pendant droplet into a Taylor cone.^8^ From the Taylor cone, a charged jet travels toward a grounded collector, yielding a nonwoven polymeric mat after solvent evaporation; see Scheme S1. Electrospinning enables the incorporation of chemotherapeutic drugs,^9,10^ biomolecules,^11^ and nanocarriers such as micelles,^12^ vesicles,^13^ or mesoporous silica nanoparticles^14,15^ into biocompatible polymers, including polycaprolactone (PCL),^16^ polylactic acid (PLA),^17^ poly­(vinyl alcohol) (PVA),^18^ or poly­(ethylene oxide) (PEO).^19^ It is important to mention that for localized drug release applications, a precise control of the final NF diameter is particularly important, as it determines encapsulation efficiencies and the capacity to host active species of different sizes. The NF diameter is governed by the interplay of solution parameters (polymer concentration, viscosity, molecular weight, solvent system),^20–24^ process parameters (voltage, flow rate, collector distance),^25–28^ and environmental parameters (temperature, humidity),^29,30^ as is summarized in Table S1. In this context, as the final nanofiber diameter results from the combined action of these mentioned variables, it is highly desirable to develop mathematical expressions that predict the fiber size as a function of key processing conditions, as it would allow a rational design of nanofiber-based delivery platforms prior to the electrospinning process.

Among numerous bioactive compounds of interest to be incorporated into electrospun NFs for localized drug release applications, ferulic acid (FA) attracts considerable attention owing to its antioxidant, photoprotective, anti-inflammatory, and wound-healing properties,^31,32^ being an important compound in *Argania spinosa*, a flowering plant species originally from the north of Morocco that has been used for centuries in traditional medicine.^33,34^ Argan extract is rich in numerous bioactive compounds such as polyphenols, tocopherols, sterols, squalene, triterpene alcohols, and monounsaturated fatty acids, which collectively contribute to its biological activity.^31,35^ FA exhibits an amphiphilic character, with both hydrophilic (hydroxyl and carboxyl) and hydrophobic (aromatic ring) moieties, which facilitates its incorporation into polymeric matrices and its subsequent release in aqueous media.^34^ Due to its antioxidant, photoprotective, anti-inflammatory, antibacterial, and wound-healing activities, FA has emerged as a promising candidate in polymeric delivery systems for localized drug release applications.^36–38^ More broadly, electrospinning belongs to the family of electrohydrodynamic atomization techniques,^39^ which also includes electrospraying and electrohydrodynamic jet printing.^40–43^ These technologies have attracted considerable attention in recent years due to their versatility for fabricating micro- and nanostructured materials not only in blend electrospinning but also for coaxial and other complicated bulk production for biomedical, pharmaceutical, electronic, and coating applications.^44–47^ Despite the large number of studies reporting innovative applications, comparatively fewer works have focused on establishing quantitative process–structure–performance relationships capable of predicting the final material characteristics from the processing parameters. Such predictive approaches are essential for improving process robustness, reproducibility, and future scale-up of electrohydrodynamic manufacturing technologies.

In this work, we have developed a three-dimensional response-surface model that provides a predictive mathematical relationship between the PCL nanofiber diameter and two key processing variables, the electrospinning flow rate (*Q*) and the DMF/DCM solvent ratio (*R*), while keeping constant the polymer mass. Using this model, the nanofiber diameter can be totally and conveniently predicted, at least, in the wide diameter range obtained (727–2925 nm), determined by scanning electron microscopy (SEM) images. After that, to establish a direct link between the processing conditions that determine nanofiber diameter and the resulting drug delivery performance, FA was incorporated into selected PCL NFs at different loadings (0.5, 2, and 6 wt %). After release analysis, we confirm that the FA release is not controlled by the nanofiber diameter, being governed by the percentage of molecules incorporated into the PCL NFs. Finally, the released investigations using three release kinetic models reveal that FA release is influenced by the flow rate used during PCL nanofiber fabrication. After discarding a total diffusive Higuchi model, we observed a release with a small diffusing contribution for nanofibers fabricated at 0.5 mL/h (Peppas–Sahlin model) and a burst release behavior (first-order kinetics) at higher flow rates. This integrated approach enables the possibility to develop a mathematical relationship between flow rate and solvent ratio to obtain different nanofiber diameters using the same polymer mass, which also provides an assessment of whether the same parameters governing nanofiber diameter also control the release behavior, providing a rational framework for designing electrospun drug delivery systems.

## Experimental Section

### Materials

Polycaprolactone (PCL) (Mw = 80,000 Da) and the solvents *N,N*-dimethylformamide (DMF) and dichloromethane (DCM) were obtained from Merck, and they were used without further purification. Water was obtained from a Millipore Milli-Q system. Ferulic acid (99.99%) was purchased from BLD Pharmtech. Phosphate-buffered saline (PBS) 0.1 M with a pH of 7.4 was supplied by Sigma-Aldrich. The eluent, HPLC-grade methanol (99.9%, MeOH), was acquired from Honeywell Chromasolv.

### Characterization

The fabricated PCL NFs were characterized by SEM (JEOL JSM 6335F, Tokyo, Japan), working at an accelerating voltage of 15 kV. After electrospinning, the samples were obtained as circular mats with diameters of approximately 12 cm. For SEM analysis, small sections with a diameter of approximately 1 cm were cut from the mats and sputter-coated with a thin gold layer using an LEICA EM ACE 200 sputter coater to minimize charging effects. Fiber diameter distributions, including the average fiber diameter and standard deviation, were determined from SEM micrographs by measuring at least 100 individual fiber segments by using ImageJ software. The viscosity of the polymeric solutions was measured by using a Brookfield Ametek low-torque viscometer.

The amount of FA released was quantified using an Agilent 1260 HPLC-DAD system equipped with a ZORBAX Eclipse Plus C18 column (100 mm × 2.1 mm i.d., 1.8 μm particle size). The mobile phase consisted of methanol as solvent B. Quantification of FA was performed using an external calibration curve constructed at a detection wavelength of 312 nm, using standard solutions with concentrations ranging from 1 to 500 μM (Figure S1A).

UV–vis spectra were recorded using a Jasco V-730 spectrophotometer in the 190–500 nm range, employing quartz cuvettes with a 1 cm optical path length and THF as the blank. The absorbance maximum at 320 nm was used to quantify the amount of FA retained in the PCL NFs after the release assay. To determine the concentration of FA remaining in the PCL NFs, a calibration curve was constructed using standard FA solutions with concentrations ranging from 1 to 100 μM (Figure S1B). When absorbance values fell outside the linear range of the calibration curve, the samples were appropriately diluted and remeasured.

### Preparation of Polymeric Solutions for Electrospinning

Initially, PCL solutions without FA were prepared by dissolving a fixed amount of polymer (1.7 g) in solvent mixtures of DMF and DCM at different weight ratios, denoted as *R* (*R* = 4, 1.5, 1, and 0.67), and processed at four different flow rates, denoted as *Q* (*Q* = 0.5, 1, 1.5, and 2 mL h^–1^). The notation used to identify the resulting PCL NFs was defined as PCL_M_R_Q, where M represents the mass of PCL in the polymeric solution and *R* and *Q* correspond to the solvent ratio and flow rate, respectively. Polymeric solutions were prepared by adding the appropriate volumes of DCM and DMF (see Table 1) to a 20 mL vial. The mixture was placed in a water bath at 45 °C under constant magnetic stirring (*ca*. 100 rpm). To avoid polymer agglomeration during dissolution, PCL was gradually added in small portions, ensuring complete dissolution before the addition of the next portion. Once the polymer was fully dissolved, the temperature was reduced to 35 °C and maintained until the electrospinning process was carried out.

**Table 1:** Composition of the PCL Solutions Employed for Electrospinning, Showing the Polymer Mass, DMF/DCM Solvent Ratio, Solvent Volumes, Total Solution Volume, and Resulting PCL Concentration

Sample	PCL (g)	DMF (g)	DCM (g)	R	DMF (mL)	DCM (mL)	Volume (mL)	PCL (mg/mL)
PCL_1.5_4_1	1.5	8	2	4	8.47	1.50	9.97	150.45
PCL_1.7_4_1	1.7	8	2	4	8.47	1.50	9.97	170.51
PCL_1.9_4_1	1.9	8	2	4	8.47	1.50	9.97	190.57

Polymeric samples containing FA were prepared by adding a defined amount of FA (0.5, 2, and 6 wt % with respect to the PCL mass) to each polymeric formulation described in Table 1. For each type of PCL nanofiber (defined by its specific flow rate and solvent ratio), three independent samples were therefore prepared, corresponding to the three FA loadings. Consequently, a total of 12 FA-loaded samples were obtained, resulting from the combination of three different PCL formulations and three FA concentrations.

The appropriate amount of FA and the corresponding volumes of solvents were added to a 20 mL vial (see Table 1), and the mixture was placed in a water bath at 45 °C under constant magnetic stirring (100 rpm). As in the FA-free formulations, PCL was added gradually in small portions to prevent polymer agglomeration. Once complete dissolution was achieved, the temperature was reduced to 35 °C and maintained until the electrospinning process was performed. To determine the amount of FA retained within the PCL NFs after the release experiment (48 h), the samples were removed from the release medium and allowed to dry to eliminate residual PBS. The dried NFs were then transferred to a 10 mL vial, and 3 or 5 mL of THF was added, depending on the initial fiber mass, to fully dissolve the polymer matrix. After complete dissolution, the FA content was quantified by UV–vis spectroscopy as described above.

### Synthesis of PCL NFs by Electrospinning

In all cases, PCL NFs were fabricated at atmospheric pressure and room temperature (23 ± 2 °C). The electrospinning experiments were carried out under ambient laboratory conditions without permanent control of relative humidity. The electrospinning setup consisted of a syringe pump, a metallic injector, two high-voltage power supplies for Taylor cone generation, and a grounded collector (Scheme S1). Each previously prepared polymeric solution was loaded into a 10 mL plastic syringe and delivered through a PTFE capillary to the injector, where the Taylor cone was formed. The injector had an outer diameter of 0.9 mm and an inner diameter of 0.6 mm, while the PTFE capillary connecting the syringe to the injector had an outer diameter of 1.6 mm and an inner diameter of 0.8 mm. A voltage in the range of 16.8–17.2 kV was applied to generate and maintain a stable Taylor cone. The positive electrode was connected to the injector, whereas the negative electrode was connected to the collector, which consisted of a stainless-steel flat platform covered with aluminum foil for fiber deposition (Scheme S1). The electrospinning was carried out for 1 h in all cases, resulting in the formation of a polymeric mat with an approximate diameter of 12 cm on the aluminum foil. The injector–collector distance was fixed at 15 cm for all the experiments. After electrospinning, the obtained nanofibrous mats were placed in a vacuum oven and dried at room temperature for 12 h to remove any residual solvent.

### Release Kinetics of Ferulic Acid

To investigate the release kinetics of FA, the fabricated PCL NFs were cut into circular discs with a diameter of 35 mm and placed in 100 mL polypropylene flasks. Subsequently, 20 mL of release medium (10 mM PBS) was added, and the flasks were placed on an orbital shaker operating at 100 rpm.

Aliquots of the release medium were collected at predetermined time points corresponding to 0.25, 0.5, 1, 1.5, 2, 3, 4, 5, 6, 7, 8, 24, 30, and 48 h. For samples containing 6 wt % FA, 100 μL aliquots were withdrawn, whereas for samples containing 2 and 0.5 wt % FA, 200 μL aliquots were collected. After each sampling, the same volume of fresh PBS was added to the release medium to maintain a constant total volume throughout the experiment. The concentration of FA in the collected aliquots was determined using an HPLC-DAD system following a predefined analytical sequence. The chromatographic analysis was performed using 100% methanol as the mobile phase at a flow rate of 0.5 mL min^–1^, an injection volume of 5 μL, and a column temperature of 30 °C. The system pressure was maintained between 50 and 60 bar. To determine the amount of FA remaining within the PCL nanofibers after completion of the release experiment (48 h), the nanofibrous mats were removed from the release medium and allowed to dry to eliminate residual PBS. The dried samples were then transferred to a 10 mL vial, and 3 or 5 mL of THF was added, depending on the initial fiber mass, to fully dissolve the polymer matrix. After complete dissolution, the FA content was quantified by UV–vis spectroscopy as described in Section Preparation of Polymeric Solutions for Electrospinning.

### Statistical Analysis

The amount of FA released per mg of NFs and the release percentage are represented as the mean ± standard deviation of three replicates.

## Results and Discussion

### Fabrication of PCL NFs with Different Fiber Diameters

For the electrospinning process, PCL was dissolved in four different mixtures of DMF and DCM. This solvent composition was defined by the solvent mass ratio *R* (DMF/DCM, w/w). In order to identify suitable conditions for obtaining smooth, homogeneous, and bead-free nanofibers, an initial optimization step was carried out by varying the amount of polymer in the spinning solution. Based on previous reports and preliminary experiments, three different PCL masses (1.5, 1.7, and 1.9 g) were evaluated,^5,9^ keeping the total solvent mass constant at 10 g. During this initial screening, the electrospinning flow rate was fixed at 1 mL h^–1^, and the solvent ratio was set to *R* = 4, allowing the effect of polymer concentration on fiber formation to be initially assessed. This approach enabled the identification of a suitable concentration for producing uniform nanofibers without bead formation. As described in the Experimental Section, the samples obtained in this stage were denoted as PCL_M_R_Q, where M corresponds to the mass of PCL used in the polymeric solution, *R* to the DMF/DCM solvent mass ratio, and *Q* corresponds to the applied flow rate during electrospinning. Table 1 summarizes the composition of the polymeric solutions employed in this initial study, including the amount of PCL, solvent composition, total solution volume, and the resulting polymer concentration (expressed as mg of PCL per mL of solution).

Figure 1 shows representative SEM images of PCL nanofibers using 1.5, 1.7, and 1.9 g of polymer at a fixed ratio of 4 (*R* = 4) and a constant flow rate of 1 mL h^–1^. As can be observed, 1.5 g of PCL led to the formation of inhomogeneous fibers with a significant presence of beads, indicating insufficient chain entanglement in the polymer solution. In contrast, samples prepared with 1.7 and 1.9 g of PCL resulted in smooth and well-defined nanofibers, with a more homogeneous morphology. Among these, the formulation containing 1.7 g of PCL exhibited the most uniform fiber distribution and was therefore selected for all subsequent experiments. Based on these results, the effect of additional processing parameters on nanofiber morphology was investigated using a fixed PCL mass of 1.7 g.

**Figure 1:**
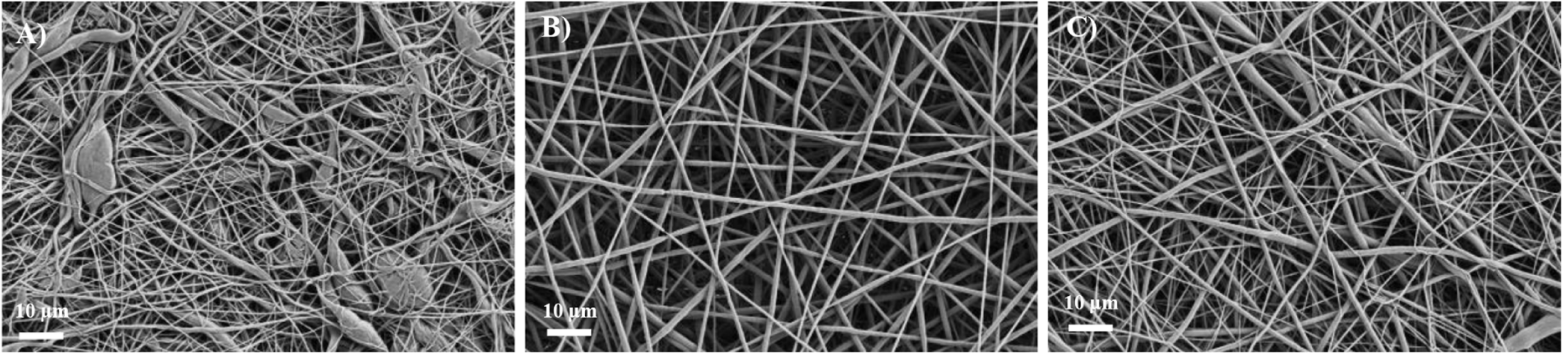
Representative SEM images of PCL nanofibers electrospun at a fixed solvent ratio (*R*, DMF/DCM = 4) and a constant flow rate of 1 mL h^–1^using different polymer masses: (A) 1.5 g, (B) 1.7 g, and (C) 1.9 g of PCL.

After that, the investigated parameters included the flow rate applied during electrospinning, the amount of polymer delivered through the Taylor cone, the solvent ratio used for solution preparation, and the resulting viscosity of the polymeric solutions. Table 2 summarizes the sample nomenclature, PCL mass, DMF/DCM ratio, total solution volume, polymer concentration, amount of polymer delivered through the Taylor cone over 1 h at different flow rates (0.5, 1.0, 1.5, and 2.0 mL h^–1^), and the corresponding viscosity values. It is important to mention that, due to the different densities of DMF (0.944 g mL^–1^) and DCM (1.33 g mL^–1^), variations in the DMF/DCM ratio led to slight differences in the total solution volume and, consequently, in the effective polymer concentration. For this reason, Table S2 provides a detailed description of the solvent volumes used at each *R* value, together with the resulting total volume and polymer concentration for each formulation.

**Table 2:** Sample Notation, Mass of Polymer, *R* Value, Total Volume, Concentration of PCL, Amount of PCL That Crosses the Taylor Cone in 1 h at Different Flow Rates (0.5, 1, 1.5, and 2 mL h^–1^), and Viscosity of Each Prepared Sample

PCL_M_R	PCL (g)	R	Volume (mL)	PCL (mg/mL)	Q = 0.5	Q = 1	Q = 1.5	Q = 2	*η* (cP)
PCL_M1.7_R4	1.7	4	9.98	170.36	85.18	170.36	255.54	340.72	1628
PCL_M1.7_R1.5	1.7	1.5	9.36	181.55	90.78	181.55	272.33	363.10	2572
PCL_M1.7_R1	1.7	1	9.06	187.72	93.86	187.72	281.58	375.44	2834
PCL_M1.7_R0.67	1.7	0.67	8.75	194.31	97.16	194.31	291.47	388.62	3096

In the next stage, the combined influence of the electrospinning flow rate (*Q*) and the solvent ratio (*R*) on the average nanofiber diameter was systematically evaluated. To this end, a three-dimensional response-surface analysis was employed to describe the simultaneous effect of both parameters on the resulting PCL nanofibers. Prior to this analysis, the morphology and diameter distributions of the electrospun fibers were examined by SEM images. Representative micrographs of PCL nanofibers obtained at flow rates of 0.5, 1.0, 1.5, and 2.0 mL h^–1^, each prepared using different solvent ratios (*R* = 4, 1.5, 1, and 0.67), are shown in Figures 2 and 3 includes representative SEM images for PCL nanofibers obtained at the lowest, 0.5 mL/h, and the highest, 2 mL/h, flow rates prepared using different solvent ratios (*R* = 4, 1.5, 1, and 0.67). Representative SEM images for PCL nanofibers obtained at 1.0 and 1.5 mL/h are included in Figures S2 and S3, respectively.

**Figure 2:**
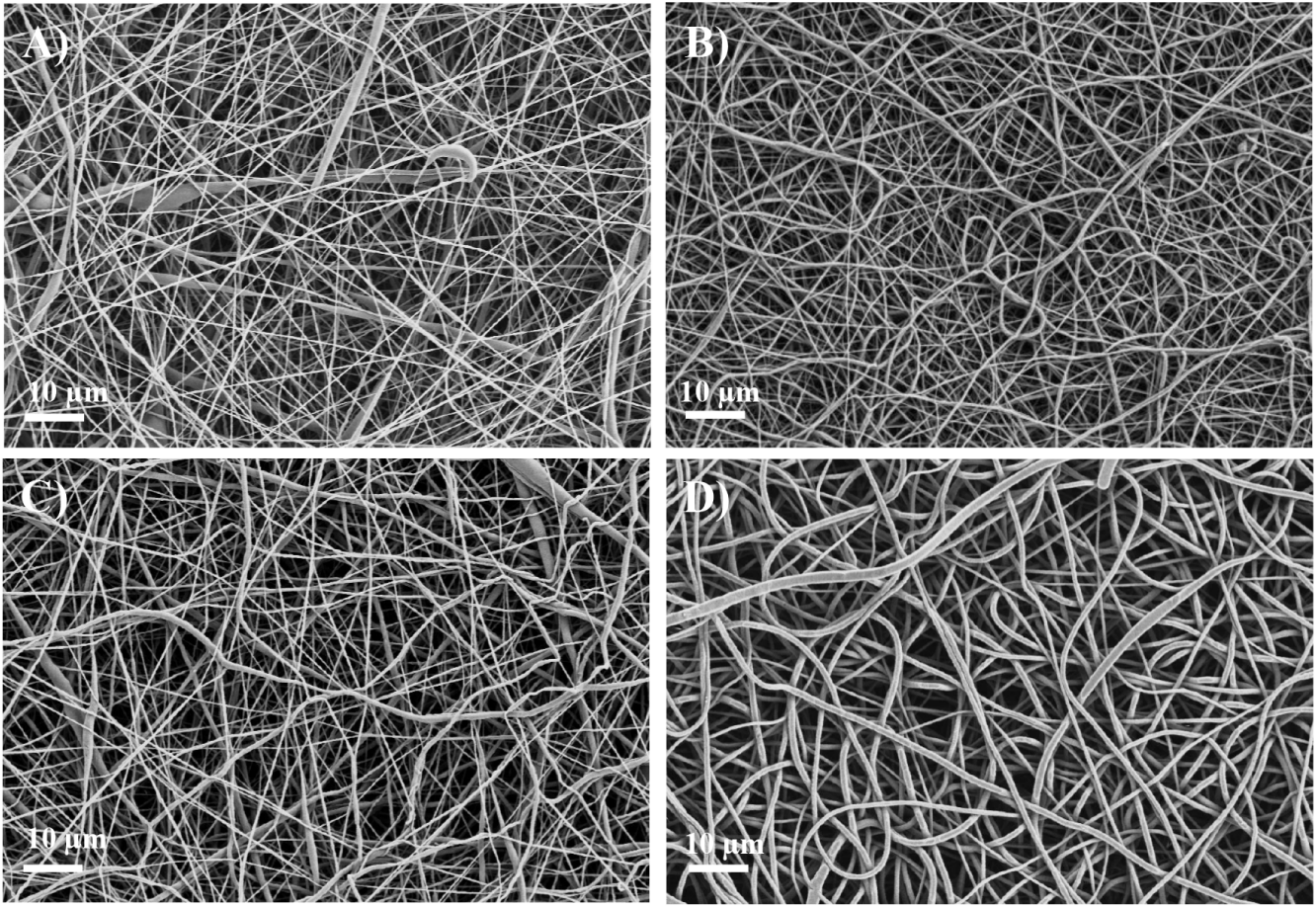
Representative SEM images of the PCL NFs obtained at a fixed PCL mass of 1.7 g and *R* (DMF/DCM) = 4 at different flow rates: A) 0.5 mL/h, B) 1 mL/h, C) 1.5 mL/h, and D) 2 mL/h.

**Figure 3:**
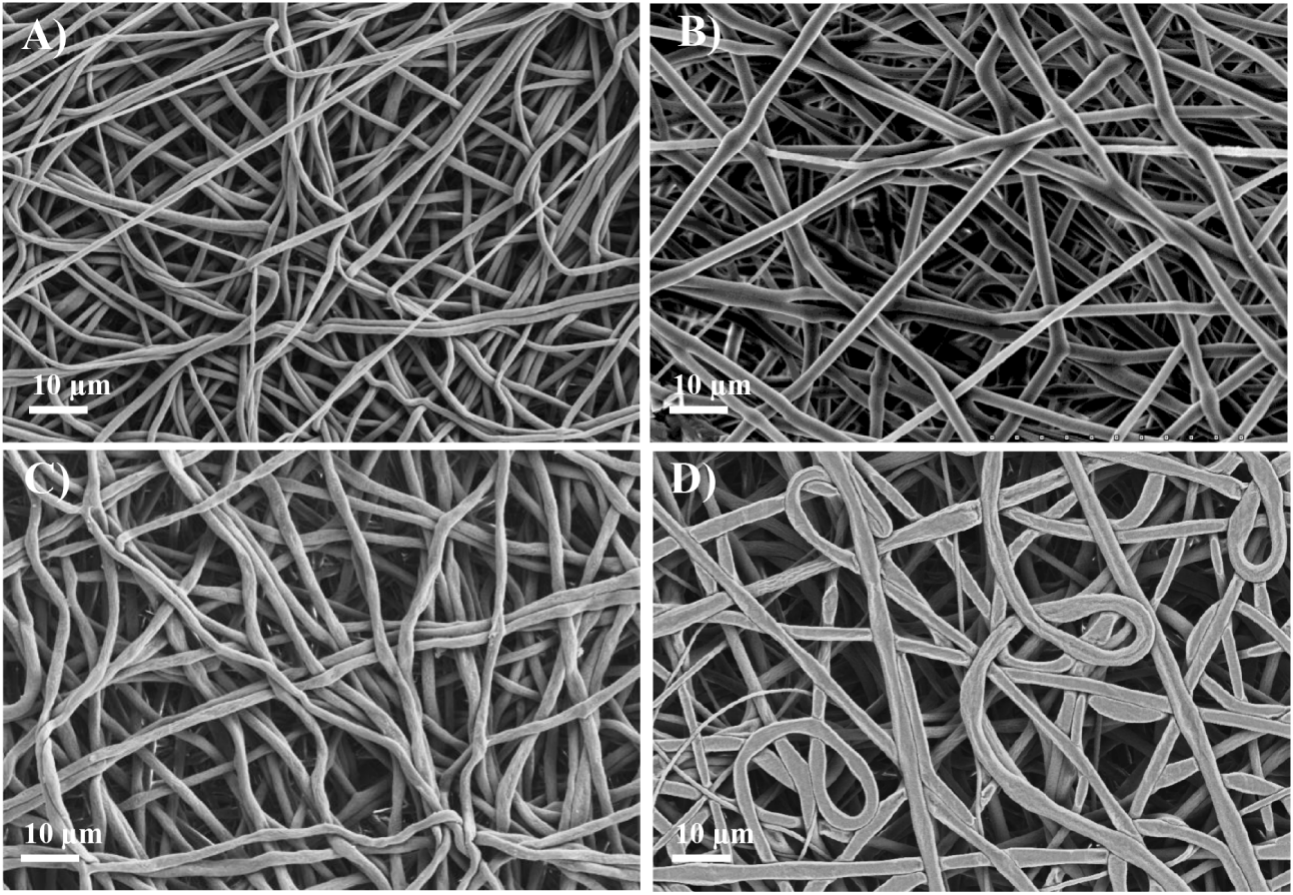
Representative SEM images of the PCL NFs obtained at a fixed PCL mass of 1.7 g and *R* (DMF/DCM) = 0.67 at different flow rates: A) 0.5 mL/h, B) 1 mL/h, C) 1.5 mL/h, and D) 2 mL/h.

As can be observed, smooth, homogeneous, and bead-free nanofibers were obtained in all cases, confirming the robustness of the selected electrospinning conditions across the explored parameter space. The corresponding fiber diameter distributions, obtained from all these SEM images, including average diameter values and standard deviations, are presented in Figures S4–S7, while the numerical values of these parameters, used for the three-dimensional representation, namely, average fiber diameter, as well as the amount of polymer delivered through the Taylor cone, flow rate (*Q*), and solvent ratio (*R*), are summarized in Table S3. As can be extracted from the SEM images, and for the corresponding size distribution, the average nanofiber diameter increased as the flow rate applied during the electrospinning process also increases for each of the individual solvent ratios used. Indeed, it is also observed that when the *R* value decreased from *R* = 4 to *R* = 0.67, i.e., as the amount of CH_2_Cl_2_ is higher, the average nanofiber size also increases, even with a stronger tendency. For this reason, with the aim to obtain a mathematical expression that allows us to link both parameters with the observed trends, the average nanofiber diameter (Z, nm) was modeled as a function of these two independent variables, the flow rate (*Q*, mL h^–1^) and the solvent ratio (*R* = DMF/DCM, w/w).

Among the tested mathematical analyses, the best compromise between simplicity and goodness of fit was obtained using a logarithmic dependence on *Q* combined with an inverse dependence on R, leading to the following empirical expression:

1 Z(Q,R)=a(R)ln(Q)+b(R)

with

2 $$a\left(R\right)=\frac{521.7}{R}+397.3,,b\left(R\right)=\frac{943.9}{R}+709.6$$

It is evident that both fitting parameters, *a­(R)* and *b­(R)*, increase as the value of *R* decreases, which is consistent with the previous experimental observation derived from the SEM analysis. Specifically, lower DMF/DCM ratios (i.e., higher DCM content) and the associated increase in solution viscosity (Table 2) lead to the formation of thicker PCL NFs, as clearly observed in the SEM images in Figures 2, 3, and S2 and S3, and the nanofiber size distribution included in Figures S4–S7. The resulting three-dimensional response surface, combining experimental data and the fitted model, is presented in Figure 4A. This surface represents the average nanofiber diameter as a function of both the flow rate (*Q*, mL h^–1^) and the solvent ratio (*R*). For each fixed solvent composition (*R* = 4.0, 1.5, 1.0, and 0.67), an isocurve of the form:

$$Z\left(Q,R_{k}\right)=a\left(R_{k}\right)\cdot \ln Q+b\left(R_{k}\right)$$

is plotted, allowing direct visualization of the evolution of the nanofiber diameter with increasing flow rate at a constant polymer mass (1.7 g). Interestingly, for each solvent ratio, the dependence of the nanofiber diameter on the flow rate is well-described by a logarithmic relationship, as expressed in eqs 3–6. This behavior highlights the dominant role of the flow rate in controlling fiber stretching and jet stability under fixed compositional conditions, while also reflecting the modulation imposed by solvent composition through its effect on solution viscosity and evaporation dynamics.

3 R=4.0→Z=538.1⁡ln(Q)+985.9

4 R=1.5→Z=731.1⁡ln(Q)+1280.9

5 R=1.0→Z=916.3⁡ln(Q)+1649.0

6 R=0.67→Z=1182.3⁡ln(Q)+2140.7

**Figure 4:**
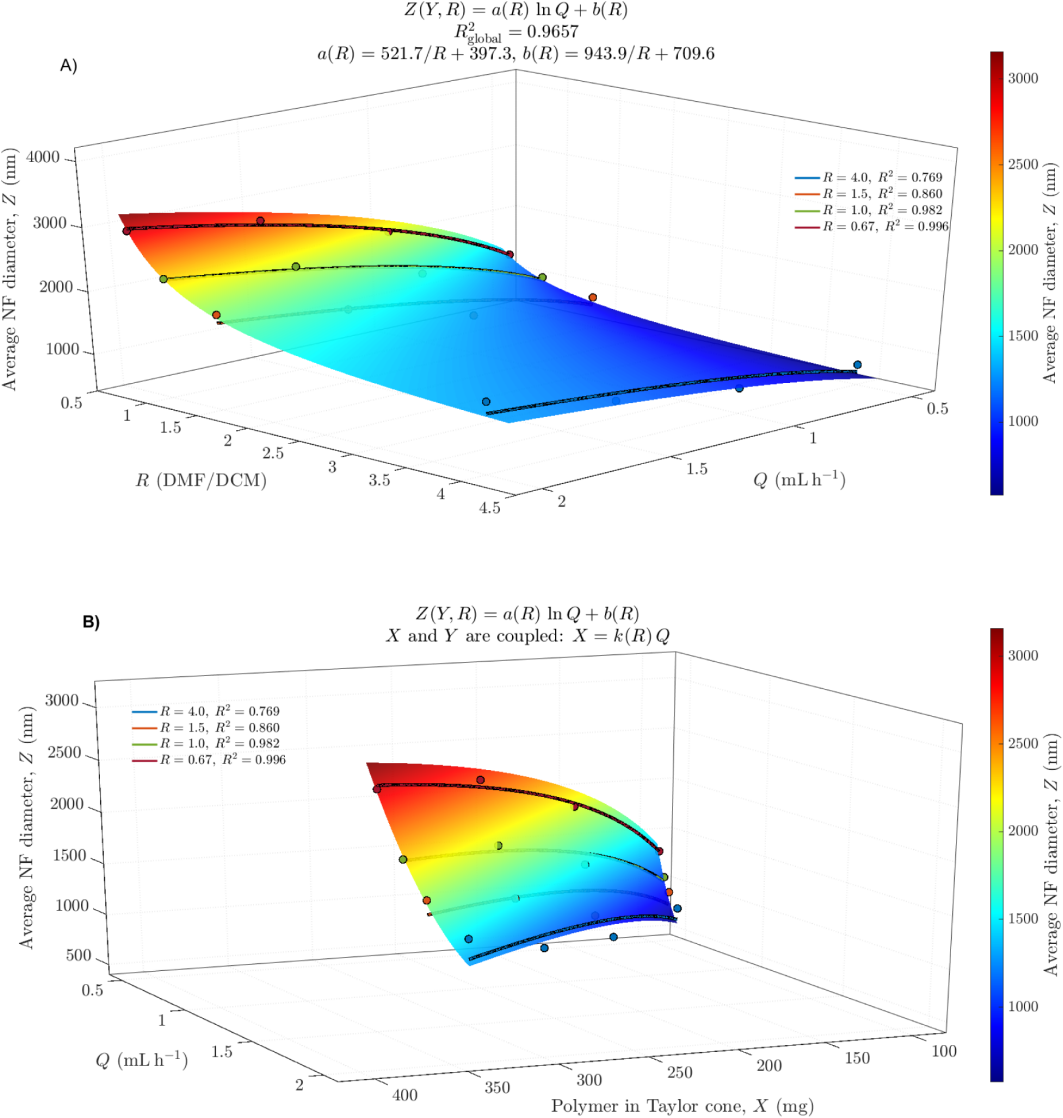
Three-dimensional response-surface model for the average diameter of PCL NFs as a function of the processing variables. A) Surface *Z*(*Q*,*R*) = *a*(*R*) · ln*Q* + *b*(*R*), where *Z* is the average nanofiber diameter (nm), *Q* is the electrospinning flow rate (mL h^–1^), and *R* is the DMF/DCM solvent ratio. Colored symbols correspond to the experimental diameters, and solid lines are the isocurves *Z*(*Q*,*R_k_*) for each solvent ratio. B) Same surface represented as a function of the polymer mass in the Taylor cone (*X*, mg) and the flow rate *Q*, where *X* and *Q* are coupled through *X* = *k*(*R*) · *Q*.

It is worth mentioning that, within the investigated range of flow rates (0.5–2.0 mL h^–1^), an approximately linear increase in the average nanofiber diameter was observed for all solvent ratios studied. This trend is clearly illustrated in Figure S8, which presents a two-dimensional representation of the average fiber diameter (including standard deviation) as a function of the flow rate for each solvent ratio. Nevertheless, although the experimental data appear nearly linear within this interval, the logarithmic formulation adopted in the model prevents nonphysical extrapolations that would arise from a purely linear dependence at higher flow rates.

From a mechanistic point of view, increasing the flow rate leads to a higher amount of polymer being delivered to the Taylor cone per unit time, thereby increasing the mass flux of the jet. Under the stable electrospinning conditions employed in this study, a higher flow rate results in a thicker electrified jet and a reduced residence time for solvent evaporation before deposition on the collector. As a consequence, thicker fibers were formed. This behavior explains the observed increase in the average nanofiber diameter (*Z*) with an increasing *Q*, which is accurately captured by the logarithmic dependence implemented in the model.

The second variable considered in the analysis is the solvent ratio, R, directly influences key physicochemical properties of the spinning solution, including viscosity, conductivity, and volatility. As shown in Table 2, decreasing the *R* value produces an increase in the DCM fraction relative to DMF, leading to a substantial increase in solution viscosity, from 1628 cP at *R* = 4 to 3096 cP at *R* = 0.67. This more viscous and less conductive jet undergoes less stretching under the applied electric field, resulting in systematically thicker nanofibers. Accordingly, the inverse relationships

a(R)∝1/R

b(R)∝1/R

accurately capture the monotonic increase in fiber diameter as the solvent ratio decreases, while maintaining a simple and physically meaningful mathematical form. Importantly, the quality of the fit is excellent, with coefficients of determination (R^2^) of 0.998 and 0.993 for *a*(*R*) and *b*(*R*), respectively. The evolution of these parameters as a function of *R* is presented in Figure S9 where the experimental data and the corresponding inverse fits are shown.

As previously mentioned, in addition to the flow rate (*Q*) and the solvent ratio (*R*), the amount of polymer passing through the Taylor cone per unit time, hereafter denoted as *X* (mg), was also evaluated on the basis of the experimental conditions summarized in Table 2. Because *X* is directly proportional to the applied flow rate (*Q*) and only weakly dependent on the solvent composition due to the relatively small variations in solution density and concentration, it cannot be considered an independent variable in the present system. Consequently, *X* can be expressed as a derived parameter that scales linearly with *Q* under the experimental conditions employed. This relationship allows the contribution of the polymer mass flux to be implicitly accounted for through the flow rate without introducing additional degrees of freedom into the model. On this basis, the amount of polymer passing through the Taylor cone per unit time can be mathematically expressed as

7 X=k(R)Q

with

8 $$k\left(R\right)=\frac{33.3}{\sqrt{R}}+154.0$$

The experimental values of the ratio *X*/*Q* exhibit a very high degree of correlation, with an R^2^ value of 0.998, indicating that this parameter is primarily governed by the solvent composition rather than by the flow rate itself. The observed inverse square-root dependence reflects the nonlinear variation of solution properties, particularly viscosity and density, with changes in the DMF/DCM ratio. The evolution of the parameter *k*(*R*) is further illustrated in Figure S9C, confirming that, for a given solvent ratio, the amount of polymer passing through the Taylor cone is effectively determined by the applied flow rate. Consequently, the parameter X, defined as the amount of polymer transported through the Taylor cone per unit time, should not be considered an independent variable in the system. Nevertheless, for the sake of clarity and visualization, Figure 4B presents the response surface describing the dependence of the average nanofiber diameter (*Z*, nm) on both *X* (polymer mass flux, mg) and the flow rate (*Q*, mL h^–1^) at the four solvent ratios investigated. This representation highlights the strong coupling between these two parameters and their combined influence on fiber diameter. Importantly, the three-dimensional response surface described by the expression

Z(Q,R)=a(R)·lnQ+b(R)

accounts for 96.6% of the total variance observed across all experimental conditions (global R^2^ = 0.966), encompassing the full set of 16 combinations of flow rate and solvent ratio evaluated. This model demonstrates that the average diameter of PCL nanofibers can be precisely tuned, using a constant amount of polymer, from approximately 727 nm (*R* = 4, *Q* = 0.5 mL h^–1^) to 2925 nm (*R* = 0.67, *Q* = 2.0 mL h^–1^) by adjusting only the flow rate and the DMF/DCM ratio in the spinning solution.

As discussed above, the strong influence of the solvent ratio is consistent with the pronounced increase in solution viscosity observed upon increasing the DCM content (Table 2). For this reason, the polymeric sample viscosity (*η*, mPa·s) was explicitly incorporated as a key process parameter governing nanofiber formation. The viscosity values for each formulation were experimentally determined (see the Experimental Section), and their dependence on the solvent ratio is presented in Figure 5A. The corresponding experimental values of average nanofiber diameter (including standard deviations) and solution viscosity for the four solvent ratios investigated are summarized in Table S4. As shown in Figure 5A, the data follows a monotonic, nonlinear trend that is well-described by the empirical relationship:

9 η(R)=−830.3⁡ln⁡R+2821.3

with an excellent coefficient of determination (R^2^ = 0.9895). Accordingly, decreasing the *R* value, i.e., increasing the DCM content in the polymeric solution, leads to a pronounced increase in viscosity, from approximately 1628 mPa·s at *R* = 4 to more than 3096 mPa·s at *R* = 0.67 (see Table 2 and Table S4). This behavior reflects the higher boiling point and stronger solvation capability of DMF compared with DCM, which promotes stronger polymer–solvent interactions and results in more viscous polymer solutions. Since viscosity is uniquely determined by the solvent composition, *η* does not represent an independent variable but rather a derived one. Consequently, the response-surface model

Z(Q,R)=a(R)ln⁡Q+b(R)

can be reformulated in terms of viscosity by substituting the functional dependence *η*(*R*) into the expressions for *a*(*R*) and *b*(*R*). The resulting three-dimensional response surface is shown in Figure 5B, where the average PCL nanofiber diameter (*Z*) is represented as a function of both the flow rate (*Q*) and the solution viscosity (*η*) for the four solvent ratios investigated (see Table S4). In this representation, the solid curves correspond to the four solvent ratios studied (*R* = 4, 1.5, 1, and 0.67). Two coupled effects can be clearly identified: (i) for a fixed solvent composition (or fixed *η*), the nanofiber diameter increases logarithmically with increasing flow rate, and (ii) for a fixed flow rate, higher viscosities (lower *R* values) lead to the formation of thicker nanofibers. The resulting response surface accurately captures the experimental behavior, yielding a global coefficient of determination of R^2^ = 0.966. According to this model, the average nanofiber diameter can be tuned from approximately 727 nm (*Q* = 0.5 mL h^–1^, *η* = 1628 mPa·s) to 2925 nm (*Q* = 2.0 mL h^–1^, *η* = 3096 mPa·s), demonstrating the strong and coupled influence of flow rate and solution viscosity on nanofiber formation.

**Figure 5:**
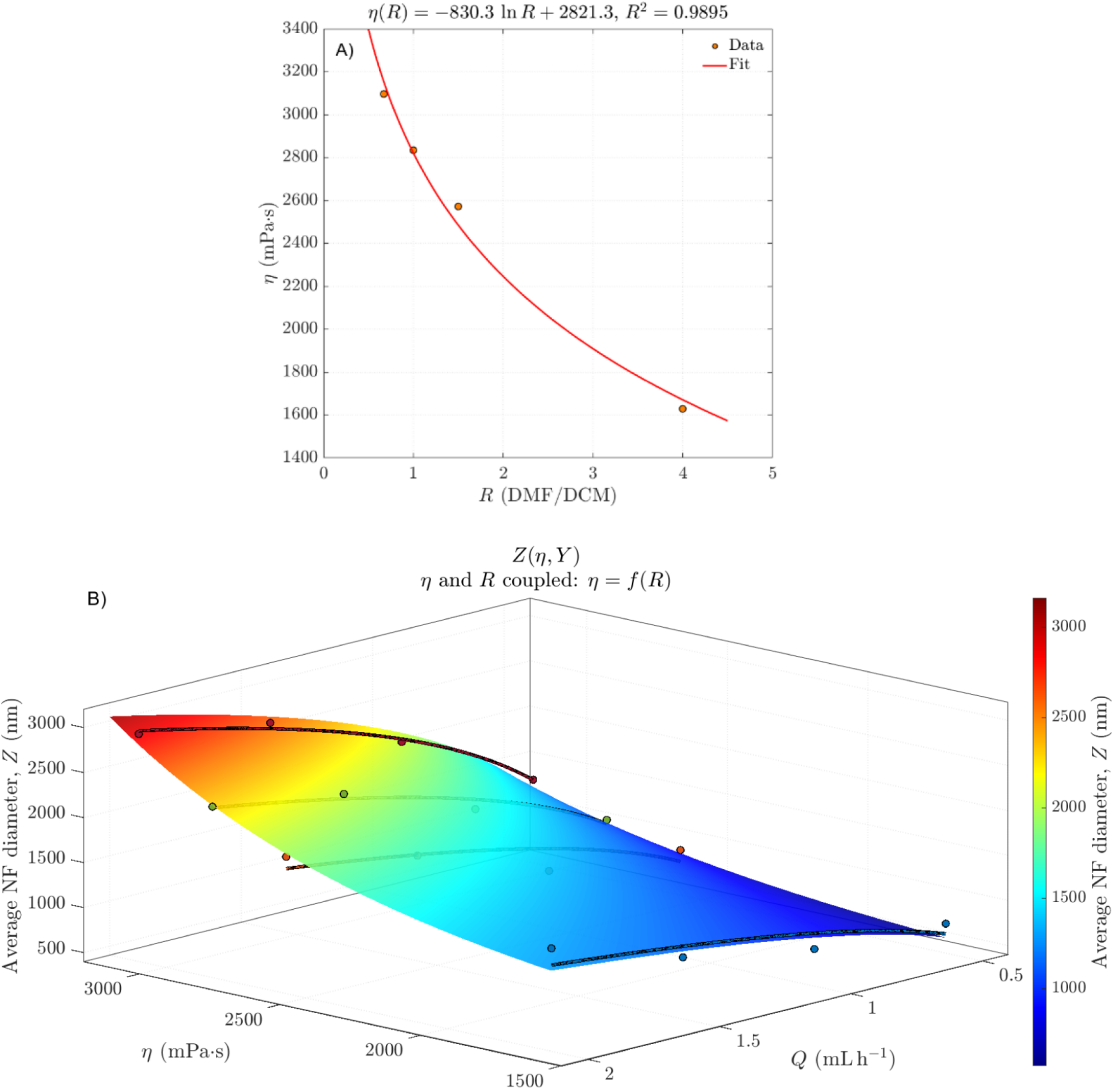
(A) Viscosity of the PCL solutions as a function of the solvent ratio *R* (DMF/DCM). Symbols correspond to the experimental data and the red line to the logarithmic fit *η­(R*) = −890.3 ln *R* + 2821.3 (R^2^ = 0.9895). (B) Three-dimensional response-surface model of the average PCL NFs diameter, *Z* as a function of the flow rate, *Q*, and the solution viscosity *η* at different solvent ratio values.

The solid curves shown in the response surface of Figure 5B correspond to the four solvent ratios investigated and clearly illustrate the coupled influence of solution viscosity and flow rate on the nanofiber diameter. Complementarily, Figure S10 presents a two-dimensional representation of the average nanofiber diameters (including standard deviations) as a function of solution viscosity for the four applied flow rates. As previously observed, for each fixed flow rate, the average fiber diameter increases monotonically with viscosity, following a nonlinear trend. At low viscosity values (*η* ≈1628 mPa·s, *R* = 4), the four curves tend to converge, indicating that under these conditions, the influence of flow rate on fiber diameter is relatively modest. In contrast, at higher viscosities (*η* > 2500 mPa·s, *R* ≤ 1.5), a pronounced divergence between the curves is observed, revealing a strong synergistic effect between viscosity and flow rate. Under these conditions, the combined effect of increased polymer viscosity and higher polymer throughput leads to a more pronounced thickening of the nanofibers than would be expected from the independent contribution of each parameter. In addition, the progressive increase in the standard deviation of the fiber diameter with both viscosity and flow rate reflects a broader size distribution for thicker fibers. This behavior is consistent with a reduced stretching efficiency of the polymer jet at high viscosities and elevated mass flow, where the balance between electrostatic forces and viscoelastic resistance becomes less favorable.

Taking it together, these results demonstrate that, for a fixed PCL mass (1.7 g), the nanofiber diameter can be precisely tuned over a wide range by jointly adjusting the flow rate and the solvent composition or equivalently the solution viscosity. In this context, representing the system in the (*η*, *Q*) space provides a particularly practical framework, as viscosity is an experimentally accessible parameter that directly links the formulation variables (DMF/DCM ratio) with the final nanofiber morphology.

The response-surface model developed above, extracted from SEM images, demonstrates that the average nanofiber diameter can be predicted across a wide size range and can be tuned by adjusting *Q* and R, keeping constant the amount of polymer. In the next section, a model molecule is used to investigate whether these same processing parameters also govern the release behavior of the encapsulated compound. To address this question, FA was incorporated into a representative set of PCL NFs previously obtained at three different loading percentages. The release behavior was systematically investigated as a function of the drug loading and fiber diameter. The last section includes the modeling of the released results obtained with three different release kinetics models, the Higuchi, the Peppas–Sahlin, and a first-order kinetic model.

### Release Kinetics of Ferulic Acid

FA is scarcely soluble in water, with a reported solubility of approximately 0.78–0.91 g L^–1^ at 25 °C. As described in the Experimental Section, the release experiments were performed in 20 mL of PBS under continuous agitation, and the release medium was periodically refreshed during sampling to maintain a sufficient concentration gradient between the nanofibers and the surrounding medium. It should also be noted that compounds exhibiting higher aqueous solubility would be expected to display faster release rates due to their greater affinity for the aqueous phase and the increased driving force for diffusion from the polymer matrix. FA was selected as a model bioactive compound because of its well-documented antioxidant, photoprotective, anti-inflammatory, antibacterial, and wound-healing properties. Furthermore, it is one of the major bioactive constituents present in *Argania spinosa* extracts. As described above, three different FA loadings (0.5, 2, and 6 wt %) were incorporated into the electrospun PCL NFs. During the release assays, the initial amount of FA incorporated into the nanofibers (*μg*_initial_), the percentage of FA released (*%_Rel_)*, and the percentage released as a function of time (*%_Rel_,_t_*) were determined using eqs 10–12, respectively.

10 $$\mu g_{initial}=\mu g_{released}+\mu g_{retained}$$

11 $${\%}_{Rel}=\frac{\mu g_{released,48h}}{\mu g_{initial}}\times 100$$

12 $${\%}_{Rel,t}=\frac{\mu g_{released,t}}{\mu g_{initial}}\times 100$$

Table 3 summarizes the name of the samples selected for the release experiments, including sample notation, as due to the polymer mass was maintained constant in all cases (1.7 g), the general nomenclature used for the release studies was PCL_R_Q_FA%, where *R* denotes the selected DMF/DCM ratio (*R* = 4 and 0.67), *Q* represents the selected electrospinning flow rate (0.5 and 1.5 mL/h), and FA is the percentage of ferulic acid relative to the polymer mass (0.5, 2, and 6%). It should be noted that, because different flow rates were employed during nanofiber fabrication, the total mass of electrospun material obtained in each case after the electrospinning process was not identical. To enable a meaningful comparison between release profiles obtained from nanofibers produced under different flow conditions, the amount of released FA was normalized to the mass of PCL nanofibers used in each experiment. Accordingly, the release results are here expressed as micrograms of FA released per milligram of PCL (μg released/mg NFs). This normalization ensures a direct and reliable comparison of release behavior across all samples. All release experiments were performed in triplicate, yielding deviations lower than 1% for the percentage of FA released and below 1 μg mg^–1^ for the normalized values. From the set of PCL nanofibers previously prepared and characterized in Section Fabrication of PCL NFs with Different Fiber Diameters, four representative formulations were selected for the kinetic release study in order to cover a broad range of fiber diameters. These samples were PCL_R0.67_Q1.5, PCL_R0.67_Q0.5, PCL_R4_Q1.5, and PCL_R4_Q0.5 (Table 3), corresponding to average fiber diameters of 2685, 1324, 1081, and 727 nm, respectively. For each of these formulations, release experiments were conducted using three different FA loadings (0.5, 2, and 6 wt %), allowing a systematic evaluation of the influence of both fiber diameter and FA content on the release kinetics.

**Table 3:** Sample Notation Used, Mass of PCL in the Polymeric Solution, *R* Value, Flow Rate during Electrospinning, and Percentage and Mass of FA Incorporated into the Polymer Solution

Sample	Mass of PCL (g)	*R*	*Q* (mL/h)	% FA in NFs	Mass of FA (mg)
PCL_R0.67_Q1.5_0.5%	1.7	0.67	1.5	0.5	8.5
PCL_R0.67_Q1.5_2%	1.7	0.67	1.5	2	34
PCL_R0.67_Q1.5_6%	1.7	0.67	1.5	6	102
PCL_R0.67_Q0.5_0.5%	1.7	0.67	0.5	0.5	8.5
PCL_R0.67_Q0.5_2%	1.7	0.67	0.5	2	34
PCL_R0.67_Q0.5_6%	1.7	0.67	0.5	6	102
PCL_R4_Q1.5_0.5%	1.7	4	1.5	0.5	8.5
PCL_R4_Q1.5_2%	1.7	4	1.5	2	34
PCL_R4_Q1.5_6%	1.7	4	1.5	6	102
PCL_R4_Q0.5_0.5%	1.7	4	0.5	0.5	8.5
PCL_R4_Q0.5_2%	1.7	4	0.5	2	34
PCL_R4_Q0.5_6%	1.7	4	0.5	6	102

Table 4 summarizes the key parameters associated with the release experiments, including the sample notation, the average nanofiber diameter (with standard deviation), the mass of PCL nanofibers used in each assay, the initial amount of FA incorporated into the fibers (calculated using eq 7), the total amount of FA released after 48 h, the corresponding percentage of FA released (calculated using eq 8), and the amount of FA released normalized to the mass of nanofibers. In the following sections, the release behavior of the PCL nanofibers is analyzed as a function of two main variables: (i) the percentage of FA incorporated into the nanofibers and (ii) the nanofiber diameter. This approach allows the individual and combined effects of drug loading and fiber morphology to be systematically evaluated.

**Table 4:** Sample Notation, Average Nanofiber Diameter (± SD), Mass of PCL Nanofibers, Initial FA Loading (eq 7), Total FA Released after 48 h, Percentage of FA Released (eq 8), and FA Release Normalized to Nanofiber Mass

Sample name	Average NFs diameter ± SD (nm)	Mass of NFs/mg	*μg*_*initial*_	*μg*_*released*_	*%*_*Rel*_	μg released/mg of NFs
PCL_R0.67_Q1.5_6%	2755 ± 218	154.7	8178.3	2508.3	54.2	16.2
PCL_R0.67_Q1.5_2%	2768 ± 277	77.9	1369	742	33.7	9.5
PCL_R0.67_Q1.5_0.5%	2714 ± 202	84.6	90.22	60.8	30.7	0.4
PCL_R0.67_Q0.5_6%	1194 ± 108	35.3	2451.7	2050.1	83.6	58.1
PCL_R0.67_Q0.5_2%	1205 ± 138	25.9	692.5	527.4	76.1	11.45
PCL_R0.67_Q0.5_0.5%	1245 ± 156	23.8	69.85	31.66	30.01	0.65
PCL_R4_Q1.5_6%	852 ± 120	98.2	6842	6519	95.3	66.4
PCL_R4_Q1.5_2%	906 ± 129	37.3	642.4	498.6	77.6	13.4
PCL_R4_Q1.5_0.5%	872 ± 140	57.1	196.81	28.9	14.7	0.5
PCL_R4_Q0.5_6%	602 ± 132	21.1	1185	1064	89.8	50.4
PCL_R4_Q0.5_2%	656 ± 96	16.3	251.6	175.7	69.8	10.8
PCL_R4_Q0.5_0.5%	675 ± 105	24.1	45.4	21	46.4	0.9

#### Influence on the Percentage of Ferulic Acid

As mentioned above, the influence of the FA content on the release behavior of PCL NFs was first investigated. To explore a wide range of fiber diameters and release profiles, two solvent ratios (*R* = 0.67 and 4) and two flow rates (*Q* = 0.5 and 1.5 mL h^–1^) were selected. Figure 6A shows the cumulative amount of FA released normalized to the mass of nanofibers, as a function of time for PCL nanofibers (±standard deviation) prepared at *R* = 0.67 and *Q* = 1.5 mL h^–1^ (PCL_R0.67_Q1.5), containing 6% (black squares), 2% (blue triangles), and 0.5% FA (red circles). Under these conditions, the measured average nanofiber diameters were 2755 ± 218 nm, 2768 ± 277 nm, and 2714 ± 202 nm, respectively (Table 4), indicating that variations in FA loading did not significantly affect fiber size. As shown in Figure 6A, an initial fast release phase is observed, followed by a slower and more sustained release for samples containing 6% and 2% FA, whereas a markedly lower release is observed for the 0.5% FA formulation. The total amount of FA released after 48 h reached 16.2, 9.5, and 0.4 μg mg^–1^ for nanofibers containing 6%, 2%, and 0.5% FA, respectively (Table 4). Although the absolute amount of released FA increases with increasing FA loading, the overall release efficiency remains moderate. Indeed, the percentage of FA released after 48 h (*%_Rel_*) was 54.2%, 33.7%, and 30.7% for 6%, 2%, and 0.5% FA, respectively (Figure S11A, Table 4). These results indicate that a significant fraction of FA remains entrapped within the PCL matrix after 48 h. Figure 6B presents the release profiles for nanofibers prepared at the same solvent ratio (*R* = 0.67) but at a lower flow rate (*Q* = 0.5 mL h^–1^), namely, PCL_R0.67_Q0.5. As in the previous case, increasing the FA content leads to a higher amount of FA released per milligram of nanofiber, reaching values of 58.1, 11.45, and 0.65 μg mg^–1^ for 6, 2, and 0.5% FA, respectively (Table 4). However, compared to the samples prepared at *Q* = 1.5 mL h^–1^, a markedly different release profile is observed. In this case, a pronounced burst release occurs within the first minutes, during which most of the incorporated FA is rapidly released. This behavior indicates that lowering the flow rate during electrospinning significantly enhances the accessibility of FA to the release medium. Interestingly, while higher FA contents increase the absolute amount of released compound, lower flow rates promote faster and more extensive release, likely due to differences in fiber morphology and internal FA distribution. This interplay between formulation and processing parameters highlights the tunability of the system for controlled release applications.

**Figure 6:**
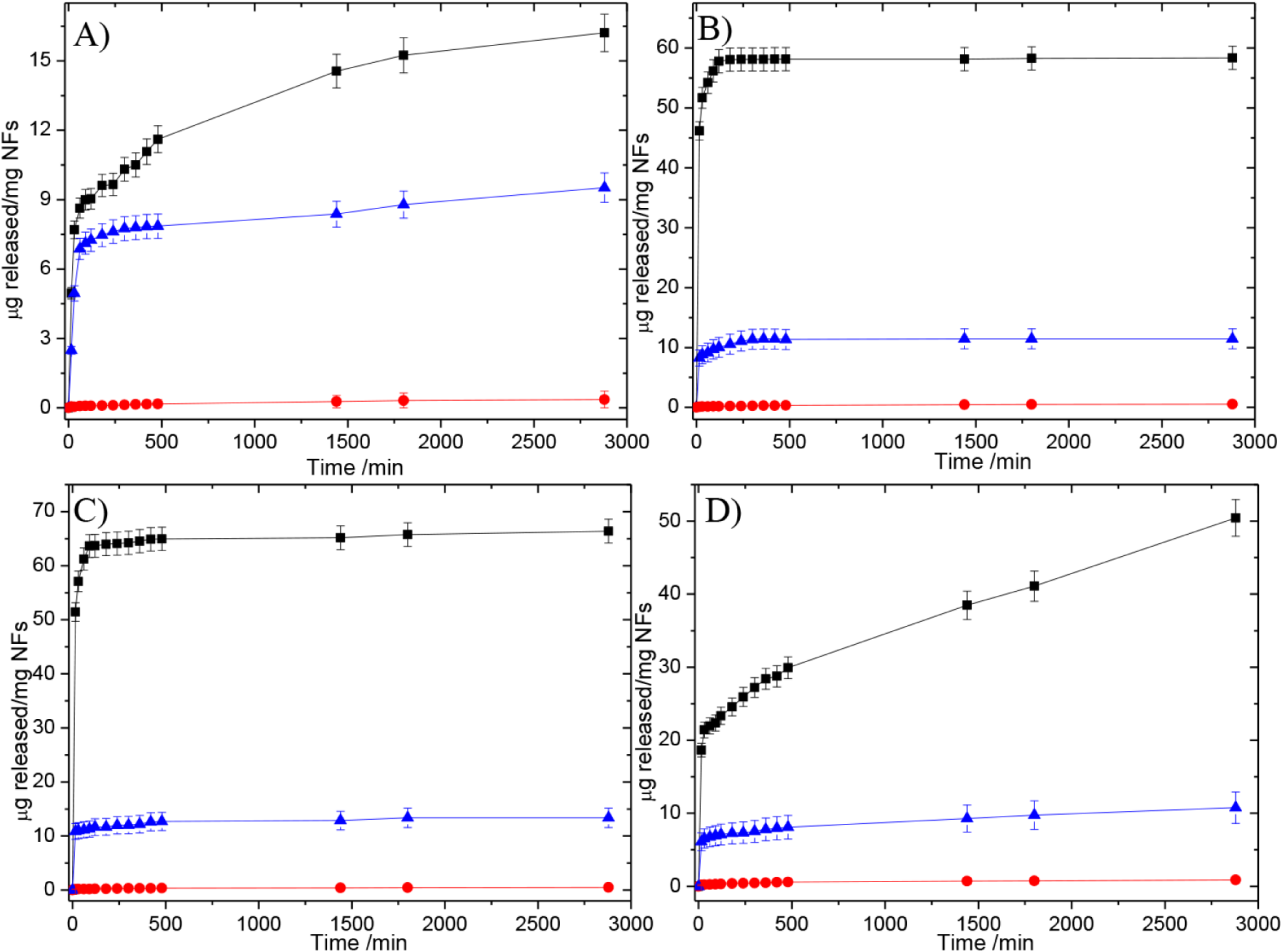
Time-dependent release profiles of ferulic acid (FA), normalized to nanofiber mass, for PCL nanofibers fabricated under different electrospinning conditions: (A) PCL_R0.67_Q1.5, (B) PCL_R0.67_Q0.5, (C) PCL_R4_Q1.5, and (D) PCL_R4_Q0.5. Curves correspond to FA loadings of 6% (black), 2% (blue), and 0.5% (red).

Figure S11B shows the percentage of FA released (*%_Rel_*, ± standard deviation) as a function of time for nanofibers containing 6, 2, and 0.5% FA prepared at a flow rate of 0.5 mL h^–1^, determined by using eq 11. Under these conditions, the release efficiencies reached 83.6, 76.1, and 30.1% for 6, 2, and 0.5% FA, respectively (Table 4), which are significantly higher than those obtained at a flow rate of 1.5 mL h^–1^. This result indicates that lower flow rates favor a more efficient release, likely due to differences in fiber morphology and internal structure. After that, the effect of the solvent ratio was further evaluated by analyzing nanofibers fabricated at *R* = 4. Figure 6C shows the release profiles for PCL_R4_Q1.5 samples containing 6 (black squares), 2 (blue triangles), and 0.5% (red circles) FA. In all cases, the amount of FA released increased with increasing FA loading. A rapid initial release was observed, followed by a plateau, indicating that most of the releasable FA was delivered during the early stages of the experiment. After 48 h, the released amounts reached 66.4, 13.4, and 0.5 μg mg^–1^ for 6, 2, and 0.5% FA, respectively (Table 4), corresponding to *%_Rel_* values of 95.3, 77.6, and 14.7% (Figure S11C). Finally, Figure 6D presents the release profiles for PCL_R4_Q0.5 samples at the three different percentage loadings. In this case, an initial burst release was again observed, followed by a slower release phase up to 48 h. The total amount of FA released reached 50.4, 10.8, and 0.9 μg mg^–1^ for 6, 2, and 0.5 FA, respectively, corresponding to *%_Rel_* values of 89.8, 69.8, and 46.4% (Figure S11D and Table 4). These results further confirm that both solvent ratio and flow rate play a decisive role in controlling FA release from PCL nanofibers. In particular, higher FA contents and lower flow rates lead to higher *%_Rel_* values, whereas formulations containing 0.5% FA consistently exhibit limited release. This behavior highlights the strong coupling between fiber morphology, drug distribution, and release kinetics.

#### Influence on the PCL NF Thickness

After evaluating the influence of FA loading on the release behavior, the effect of nanofiber thickness on FA release was subsequently investigated. For this purpose, samples containing the same FA content (6%) but different nanofiber diameters were compared. Figure 7A shows the amount of FA released, normalized to the mass of PCL nanofibers, as a function of time for four different formulations: PCL_R0.67_Q1.5 (green triangles), PCL_R0.67_Q0.5 (blue triangles), PCL_R4_Q1.5 (black squares), and PCL_R4_Q0.5 (red circles). These samples were selected to cover a broad range of fiber diameters while maintaining a constant FA content. The corresponding average nanofiber diameters, determined by SEM analysis, were 2755 ± 218 nm for PCL_R0.67_Q1.5, 1194 ± 108 nm for PCL_R0.67_Q0.5, 852 ± 120 nm for PCL_R4_Q1.5, and 602 ± 132 nm for PCL_R4_Q0.5 (Table 4). This set of samples therefore enables a direct evaluation of the influence of nanofiber thickness on FA release behavior, independently of FA loading. As observed in Figure 7A, samples PCL_R4_Q1.5 (black squares) and PCL_R0.67_Q0.5 blue triangles), with average diameters of 852 ± 120 nm and 1194 ± 108 nm, respectively, exhibited a pronounced burst release followed by a slower release phase, reaching 66.4 and 58.1 μg of FA per mg of nanofibers after 48 h (Table 4). In contrast, PCL_R4_Q0.5 (602 ± 132 nm) (red circles) showed a more gradual release profile, reaching 50.4 μg mg^–1^ at 48 h. The thickest fibers, PCL_R0.67_Q1.5 (2755 ± 218 nm) (green triangles), exhibited the lowest release capacity, with only 16.2 μg mg^–1^ released after 48 h.

**Figure 7:**
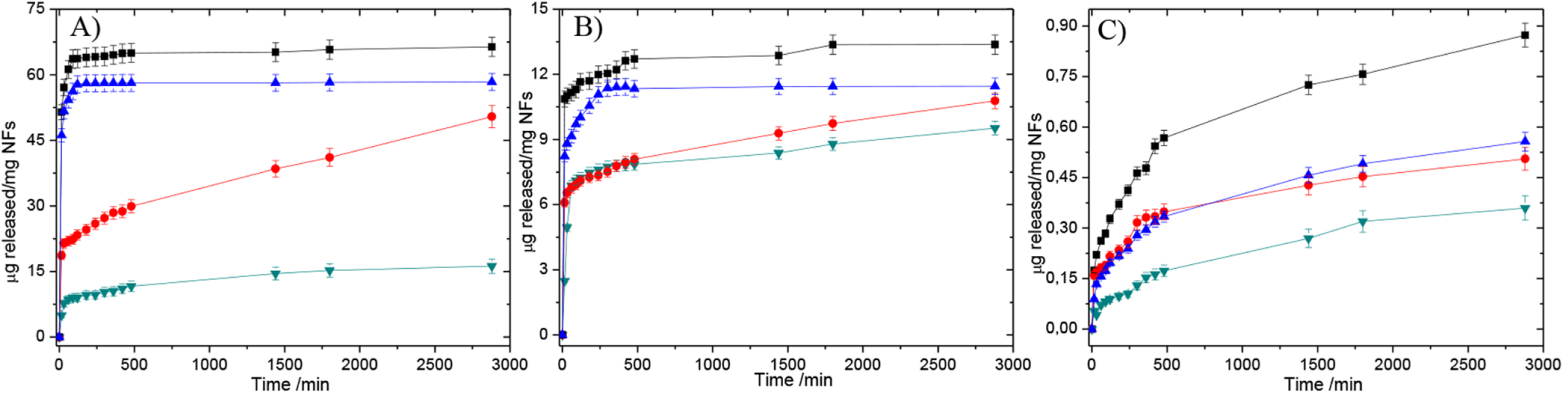
Amount of FA released divided by the mass of PCL NFs as a function of time for PCL_R0.67_Q1.5 (green triangles), PCL_R0.67_Q0.5 (blue triangles), PCL_R4_Q1.5 (black squares), and PCL_R4_Q0.5 (red circles), containing A) 6%, B) 2%, and C) 0.5% of FA.

The corresponding release efficiencies (*%_Rel_*) are shown in Figure S12A. The highest *%_Rel_* value (95.3%) was observed for PCL_R4_Q1.5 (852 ± 120 nm) (black squares), followed by PCL_R0.67_Q0.5, 1194 ± 108 nm (blue triangles) (83.6%). In contrast, PCL_R4_Q0.5 (602 ± 132 nm) (green triangles) and PCL_R0.67_Q1.5 (2755 ± 218 nm) (red circles) exhibited lower release efficiencies of 69.8 and 30.6%, respectively. These results indicate that nanofiber diameter alone does not govern the release behavior; rather, the interplay between fiber morphology and processing conditions plays a dominant role. To further validate this trend, the release behavior of nanofibers containing 2% FA was analyzed (Figure 7B). The highest FA release (13.4 μg mg^–1^) was observed for PCL_R4_Q1.5 (906 ± 129 nm) (black squares), followed by PCL_R0.67_Q0.5 (1194 ± 108 nm, 11.45 μg mg^–1^) (blue triangles), PCL_R4_Q0.5 (602 ± 132 nm, 10.8 μg mg^–1^) (red circles), and PCL_R0.67_Q1.5 (2755 ± 218 nm, 9.5 μg mg^–1^) (green triangles). A similar trend was observed for the corresponding *%_Rel_* values (Figure S12B), with PCL_R4_Q1.5 again exhibiting the highest release efficiency (77.6%, black squares). These results confirm that higher release efficiencies are not necessarily associated with smaller fiber diameters but rather with the combined effect of solvent composition and electrospinning conditions. Finally, for nanofibers containing 0.5% FA (Figure 7C), the released amounts were markedly lower, ranging from 0.4 to 0.9 μg mg^–1^, regardless of fiber diameter. The corresponding *%_Rel_* values (Figure S12C) ranged from 14.7 to 46.4%, with the highest release observed for PCL_R4_Q0.5 (602 ± 132 nm). However, due to the very low absolute amounts released, these samples were not further considered for kinetic modeling.

As mentioned, these results demonstrate that FA release is not governed solely by the nanofiber diameter. Instead, the combined effects of solvent composition and electrospinning flow rate play a dominant role in controlling both the release rate and the release efficiency. In particular, higher *R* values and lower flow rates promote more efficient FA release, while high FA loadings alone do not guarantee an enhanced release performance.

### Release Kinetics Analysis

As was mentioned in the Introduction section, three of the most representative kinetic models used in polymeric kinetics release were used to fit our kinetic data. The **Higuchi model** (eq 13) was originally developed to describe drug release from solid reservoir matrices and is still based on diffusion in a quasi-steady medium. The simplest form is expressed as

13 $$Q\left(t\right)=k_{H}\sqrt{t}$$

where *Q­(t)* is the amount of drug released per unit of mass (or surface area) of the support, *t* is the time, and *k*_*H*_ is the Higuchi constant (with units depending on the system), typically (amount) · (time)^−1/2^. The term $\sqrt{t}$ reflects that the average distance traveled by the diffusive molecules grows with the square root of time, characteristic of a purely diffusive process in a one-dimensional regime. Consequently, when the experimental data fits with this equation, it is assumed that the release is primarily controlled by the diffusion of the drug through the polymeric or fibrous matrix and that other mechanisms (dissolution of the support, swelling, degradation) have a secondary role. **Peppas and Sahlin** introduced a semiempirical model that decomposes the overall kinetics into two contributions (eq 14): one purely diffusive and the other associated with relaxation or swelling of the matrix. Its general expression is

14 $$M_{t}=k_{1}t^{m}+k_{2}t^{2m}$$

where *M_t_* is the fraction released (or accumulated mass) at time *t*, *k*_1_ and *k*_2_ are kinetic constants related to diffusion and relaxation, respectively, and *m* is the “Peppas exponent,” which provides flexibility to interpret different release rates (typical values: 0.45 ≤ *m* ≤ 1).

The first term (*k*_1_ *t^m^*) represents Fickian diffusion, that is, transport driven by a concentration gradient through a static polymer matrix, assuming negligible polymer chain relaxation and constant diffusivity. In this regime, the release rate is governed primarily by the diffusion of the solute through the polymer network. The second term (*k*_2_ *t* ^2*m*^) accounts for contributions arising from polymer relaxation, swelling, or structural rearrangements of the matrix. Therefore, when *k*_2_ is negligible after fitting, the system can be considered to behave, in practice, as a purely diffusion-controlled matrix. This model is especially useful for materials that undergo swelling or partial degradation (e.g., hydrogels, absorbent polymer fibers), where diffusive and relaxation mechanisms coexist and contribute simultaneously to the overall release process. Concerning the third release kinetic investigated, the **first-order model** is frequently obtained through eq 15:

15 $$Q\left(t\right)=Q_{\infty }\left(1-e^{-kt}\right)$$

Here, *Q*_∞_ is the maximum releasable amount (or “total charge”) and *k* is the rate constant (time^–1^). The exponential term *e* ^*–kt*^ describes how the release rate is proportional to the concentration (or amount) still retained, therefore, the slope decreases with time. When the experimental data fits this function, the release is interpreted as being limited by a desorption- or dissolution-type process (e.g., desorption of molecules weakly retained on the surface of the fibers).

For the kinetic modeling of FA release, a representative set of PCL nanofibers was selected. Specifically, three optimized nanofiber formulations with clearly differentiated average diameters were chosen, and each of them was evaluated at two relevant FA loadings (2% and 6%). This experimental design resulted in six independent release profiles (**Release 1–6**), which were used for kinetic modeling. Nanofibers prepared at *R* = 0.67 and a flow rate of 1.5 mL h^–1^ (PCL_R0.67_Q1.5) were excluded from the kinetic analysis due to their low release efficiency at all FA loadings, which resulted in 54.2% and 33.7% of release percentage for PCL NFs containing 6% and 2% (see Table 4). In addition, samples containing 0.5% FA were not considered for modeling purposes, as the amount of FA released was insufficient for reliable kinetic fitting (see Table 4). Accordingly, the release kinetic analysis was performed using the following six release profiles: **Release 1**, corresponding to PCL_R4_Q0.5_6% (602 ± 132 nm); **Release 2**, PCL_R4_Q0.5_2% (852 ± 120 nm); **Release 3**, PCL_R4_Q1.5_6% (656 ± 96 nm); **Release 4**, PCL_R4_Q1.5_2% (906 ± 129 nm); **Release 5**, PCL_R0.67_Q0.5_6% (1194 ± 108 nm); and **Release 6**, PCL_R0.67_Q0.5_2% (1205 ± 138 nm). Figure 8 shows the six release profiles corresponding to the selected PCL nanofiber formulations, revealing clearly differentiated kinetic behaviors.

**Figure 8:**
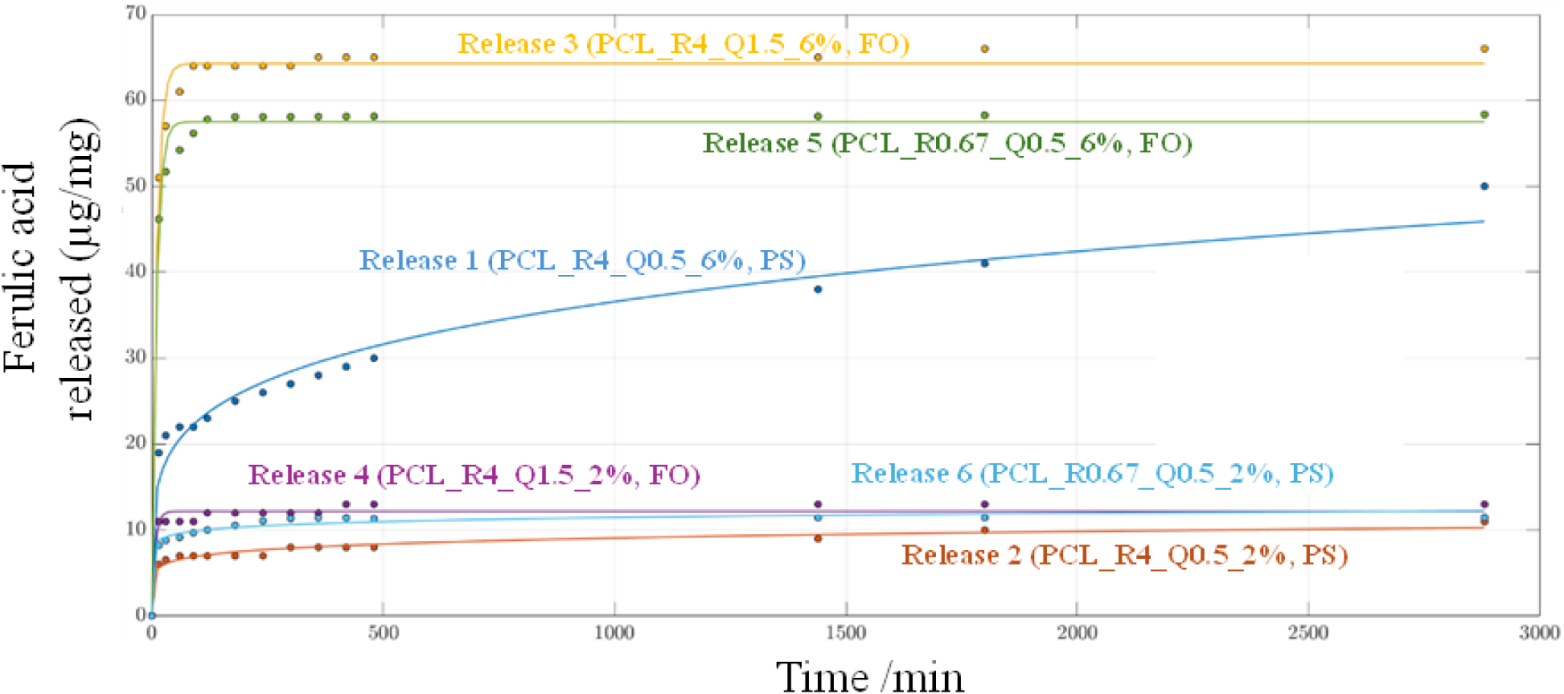
Fitting of the kinetic models to the release of FA per milligram of PCL NF for the six experiments. **Release 1** (PCL_R4_Q0.5_6%, blue line), **Release 2** (PCL_R4_Q0.5_2%, orange line), **Release 3** (PCL_R4_Q1.5_6%, yellow line), **Release 4** (PCL_R4_Q1.5_2%, purple line), **Release 5** (PCL_R0.67_Q0.5_6%, green line), and **Release 6** (PCL_R0.67_Q0.5_2%, cyan line). PS = Peppas–Sahlin; FO = first order.

This initial inspection already indicates that the release-limiting mechanisms are not identical for all samples. The kinetic parameters obtained from model fitting are summarized in Table 5.

**Table 5:** Kinetic Parameters of FA Release from PCL Nanofibers under Different Electrospinning Conditions

Release	Notation	NF diameter	R	Q (mL/h)	% FA	R^2^ Higuchi	R^2^ Peppas–Sahlin	R^2^ First order	Kinetic model
**Release 1**	PCL_R4_Q0.5_6%	602 ± 132	4	0.5	6	0.227	0.970	0.558	Peppas–Sahlin
**Release 2**	PCL_R4_Q0.5_2%	656 ± 96	4	0.5	2	–1.122	0.973	0.759	Peppas–Sahlin
**Release 3**	PCL_R4_Q1.5_6%	852 ± 120	4	1.5	6	–3.327	0.799	0.991	First order
**Release 4**	PCL_R4_Q1.5_2%	906 ± 129	4	1.5	2	–3.093	0.989	0.949	First order
**Release 5**	PCL_R0.67_Q0.5_6%	1194 ± 108	0.67	0.5	6	–2.543	0.867	0.991	Peppas–Sahlin
**Release 6**	PCL_R0.67_Q0.5_2%	1205 ± 138	0.67	0.5	2	–3.420	0.971	0.929	Peppas–Sahlin

For **Release 1** (PCL_R4_Q0.5_6%), the Peppas–Sahlin model accurately reproduces both the initial burst release and the subsequent sublinear growth observed after approximately 50–100 min. The agreement between the experimental data and model is excellent over most of the release period, with only a slight underestimation at long times. This behavior indicates that, in addition to diffusion, matrix relaxation or structural rearrangements contribute to the release process over extended time scales. A similar kinetic profile is observed for **Release 2** (PCL_R4_Q0.5_2%), despite the substantially lower total amount released (∼11 μg FA mg^–1^ NFs). The Peppas–Sahlin model again provides a good description of the sublinear release behavior, suggesting a relevant contribution from matrix relaxation. The lower value of the diffusive constant *k*_1_ indicates slower mass transport, likely associated with reduced FA loading and stronger drug–fiber interactions. In contrast, **Release 3** (PCL_R4_Q1.5_6%) is best described by a first-order kinetic model, which overlaps almost perfectly with the experimental data throughout the entire release period. This profile is characterized by a pronounced burst release during the first 10–20 min, followed by a rapid approach to a plateau (∼66.4 μg FA mg^–1^ NFs), essentially coincident with *Q*_∞_. This behavior suggests a release dominated by concentration-gradient-driven diffusion, with minimal contribution from matrix relaxation. For **Release 4** (PCL_R4_Q1.5_2%), the first-order model also provides an adequate description of the release kinetics, capturing both the initial burst and the final plateau (∼13 μg FA mg^–1^ NFs). Minor deviations observed at intermediate times (200–500 min) may arise from secondary effects such as surface desorption or microstructural heterogeneities, but their magnitude is sufficiently small to justify the use of this simplified kinetic description. This profile is consistent with a system where a large fraction of FA is readily accessible at or near the fiber surface. **Release 5** (PCL_R0.67_Q0.5_6%) exhibits a release behavior analogous to **Release 1 and 2**, with a Peppas–Sahlin fit (), a rapid initial burst followed by a matrix relaxation contribution, and a well-defined plateau (*Q*_∞_ = 57.5 μg FA mg^–1^ NFs). Finally, **Release 6** (PCL_R0.67_Q0.5_2%) is again well-described by the Peppas–Sahlin model (R^2^ = 0.971). The fitted parameters (*k*_1_ = 7.46, *k*_2_ ∼0, *m* = 0.06) indicate a release mechanism dominated by diffusion, with negligible contribution from matrix relaxation, with a total amount released (∼11 μg FA mg^–1^ NFs). After this modeling analysis, the Peppas–Sahlin model excellently fits with the experimental release data, consequent a rapid release followed by a matrix releaxation contribution, which was observed for the nanofibers systems obtained using a flow rate of 0.5 mL/h, at the two DMF/DCM ratios, *R* = 4 and *R* = 0.67. However, a fast burst release, following first-order kinetics, was observed for nanofibers fabricated at a flow rate of 1.5 mL/h. This tendency can be explained considering that at lower flow rates (0.5 mL/h), the molecules of FA can be incorporated into the PCL NFs in a more diffusive and deeper manner, thus fitting with a Peppas–Sahlin model with an initial fast release followed by a matrix relaxation. However, when the flow rate is increased (1.5 mL/h), the molecules of FA are not able to diffuse through the NFs, being preferably located on the PCL NFs surface, thus following first-order kinetics.

The global analysis reveals that the processing parameters determine the nanofiber diameter, with the possibility of developing response-surface models (*Q* and *R*), which also control the internal distribution of FA within the polymeric matrix and, consequently, the release mechanism. As was previously mentioned, at low flow rates (*Q* = 0.5 mL h^–1^), regardless of the solvent ratio, the slower jet formation allows FA molecules to diffuse deeper into the fiber interior during electrospinning, leading to a Peppas–Sahlin release profile in which an initial burst phase is followed by a matrix-relaxation contribution. In contrast, at higher flow rates (*Q* = 1.5 mL h^–1^), the rapid jet displacement limits FA penetration, resulting in a preferential surface localization and a first-order burst release. Notably, the nanofiber diameter does not dictate the release behavior, as fibers of similar diameter fabricated under different *Q* and *R* conditions exhibited markedly different kinetic profiles (Table 5). This observation indicates that the response-surface model, while accurately predicting the average nanofiber diameter, captures only one dimension of the structural outcome of electrospinning. The same processing variables simultaneously govern microstructural features, such as internal porosity, drug distribution depth, and polymer chain packing, which are not reflected in the mean diameter but critically influence drug release. Therefore, the response-surface framework should be viewed not merely as a diameter predictor but as a tool for selecting processing windows that simultaneously control both fiber morphology and drug delivery performance.

It should be emphasized that the predictive model developed in this work was established using FA as a model bioactive compound and PCL with a molecular weight of 80 kDa. Therefore, the relationships identified between electrospinning parameters, nanofiber diameter, and release behavior should be interpreted within the context of this specific polymer–drug system. While the diameter model is expected to remain applicable to similar PCL-based formulations, release kinetics may vary for compounds with different physicochemical properties or for polymers with different molecular weights, requiring validation and recalibration under the corresponding experimental conditions. Although the present study was conducted using conventional blend electrospinning, the process–structure–performance framework developed here could also be extended to advanced electrospinning configurations, such as coaxial, side-by-side, and high-throughput electrohydrodynamic manufacturing systems.

## Conclusions

In conclusion, a three-dimensional response-surface model was developed that mathematically predicts the average diameter of electrospun PCL nanofibers (global R^2^ = 0.966) as a function of the electrospinning flow rate and the DMF/DCM solvent ratio, using a constant polymer mass. As an important innovation, the model reveals a logarithmic dependence on the flow rate and an inverse dependence on the solvent ratio, enabling the controlled fabrication of nanofibers spanning from 727 to 2925 nm. Importantly, the same processing parameters that govern nanofiber diameter were found to simultaneously control the drug release behavior after the incorporation of a model molecule. The release was strongly dependent on the FA loading, with higher FA contents yielding greater release efficiencies in all cases, whereas no direct correlation with the nanofiber diameter was observed. Kinetic analysis demonstrated that the Higuchi diffusion model does not apply to these systems, compared with other reported nanofibers. Instead of that, nanofibers fabricated at low flow rates followed a Peppas–Sahlin model, consistent with an initial burst release followed by matrix-relaxation-mediated transport, while those fabricated at higher flow rates exhibited first-order kinetics, indicative of surface-dominated desorption. We hypothesize that the observed flow-rate-dependent kinetic behavior is attributed to differences in the depth of FA incorporated during electrospinning. Compared with other reported investigations for solely nanofiber fabrication, our findings establish that the included response-surface model serves a dual purpose; it enables a rational prediction of nanofiber dimensions and, simultaneously, the selection of processing conditions to yield a desired release profile. This integrated approach provides a practical framework for a tailored and controlled design of electrospun polymeric nanofibers for localized drug delivery applications. Future studies will focus on extending this approach to other bioactive compounds, polymer matrices, and advanced electrospinning configurations.

## Supplementary Material



## Notes

Schematic representation of the electrospinning process used for the fabrication of PCL NFs, influence of several parameters during the electrospinning process, different conditions used during the fabrication of PCL NFs, average NF diameter obtained under different solvent mixtures, solvent flow used, release kinetic fitting parameters, calibration strength lines of FA in water and THF, SEM images of PCL NFs with nanofiber diameter distribution, 2D graphical representations, and percentage of FA released (PDF)
