# Supplementary material for: Three-Dimensional Response-Surface Models to Predict the Diameter of Electrospun Polycaprolactone Nanofibers: Application in the release of ferulic acid
Source: ACS Omega. 2026 Jul 24;11(31):46252–67. doi: 10.1021/acsomega.6c03403 (PMC13470713; doi:10.1021/acsomega.6c03403)
Supplement: Supplementary file 1 [file ao6c03403_si_001.pdf]

Supporting Information

Three-dimensional response-surface models to predict the diameter of electrospun polycaprolactone nanofibers. Application in the release of ferulic acid.

Felipe Lestón-Cabeo,[a] Nuria Muñoz-Flores,[a] Melissa Olmedo-Navarro,[a] Francisco Arrabal-Campos,[a*] Ignacio Fernández,[a] Rafael Contreras-Cáceres.[a*]

[a] Department of Chemistry and Physics, Research Centre CIAIMBITAL, University of Almería, Almería 04120, Spain

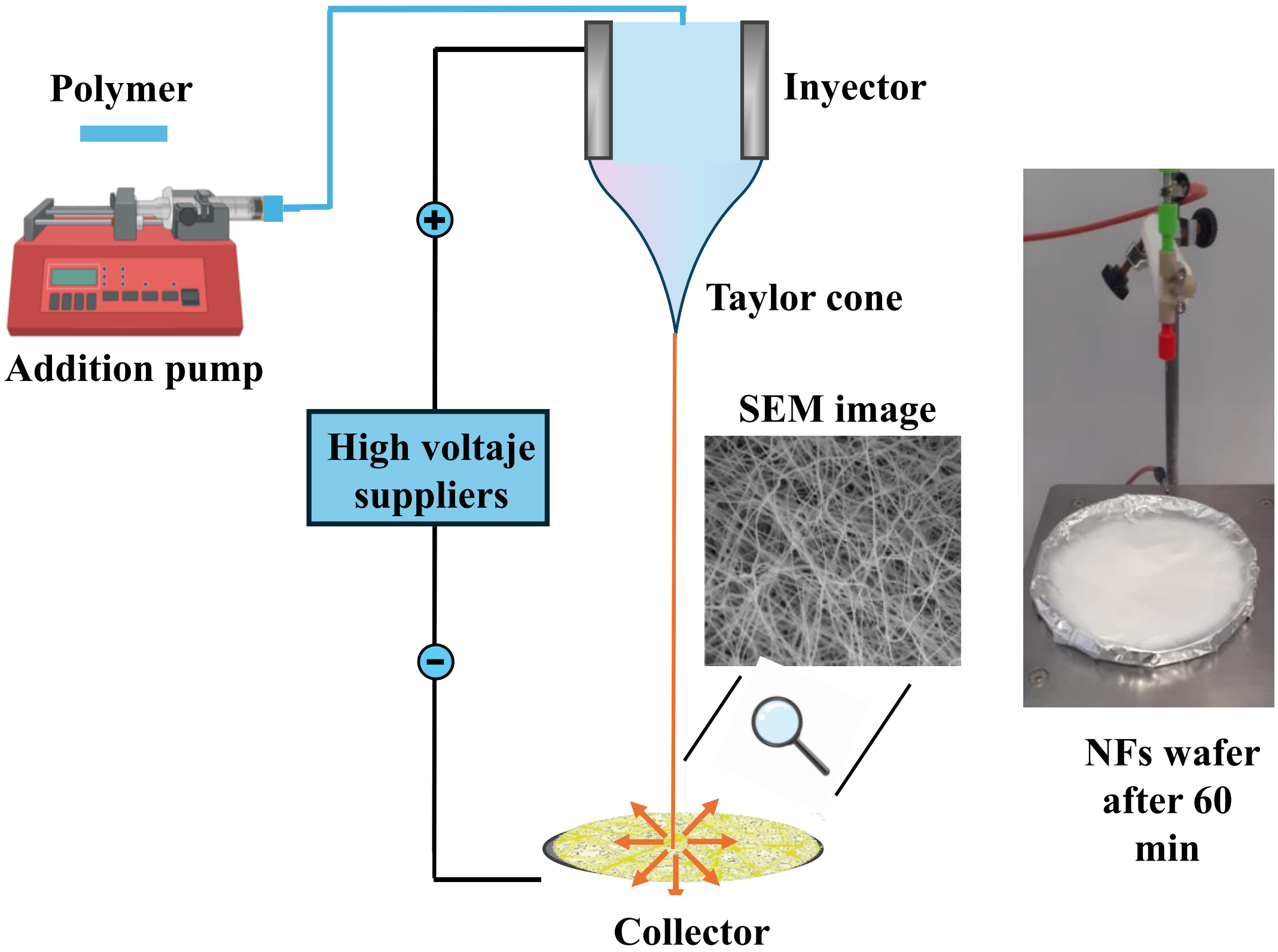

Scheme S1. Schematic representation of the electrospinning process used for the fabrication to the PCL NFs.

Table S1. Influence of solvent, process and environmental parameters in the synthesis of polymeric nanofibers by electrospinning.

**Parameter**	**Influence in the electrospinning process**
Polymer concentration	High: Unstable flow into the injector<br>Low: The polymeric chains break before reach the collector generating beads.
Viscosity	High: Crosslinking between polymeric chains.<br>Low: Beads formation.
Surface tension	High: Beads formation<br>Low: Uniform and homogeneous NFs.
Conductivity	High: Higher charge motion. Higher polymer stretching. Thinner NFs.<br>Low: Lower chrge motion. Lower polymer stretching. Thinner NFs.
Solvent volatily	High: The solvent doest not evaporate from the NFs.<br>Low: NFs drying in the injector.
Voltage	High: Thick NFs, jet multispliting. Beads generation.<br>Low: Instable Taylor cone.
Flow rate	High: Thicker NFs, higher polydispersity, bigger beads (in case they appear)<br>Low: NFs need more time for solvent evaporation
Injector-collector distance	High: Higher NFs elongation, and thinner.<br>Low: Low time to solvent evaporation. NFs are wet
Temperature	High: Decrease in NFs diameter<br>Low: Increase in NFs diameter
Humedad	High: High porosityPresencia de poros.<br>Low: Solvent drying due to spontaneous evaporation

Table S2. Sample notation, percentage of solvents, R value, total volume, concentration of PCL, amount of PCL that cross the Talor cone in 1 hour at the different flow rates (0.5, 1, 1.5 and 2 mL/h), and viscosity of each prepared sample.

Sample notation	% (w/w) DMF	% (w/w) DCM	R value	mL DMF	mL DCM	Volume (ml)	PCL Conc (mg/mL)
PCL_M1.7_R4	80	20	4	8.47	1.50	9.98	170.36
PCL_M1.7_R1.5	60	40	1.5	6.36	3.01	9.36	181.55
PCL_M1.7_R1	50	50	1	5.30	3,76	9.06	187.72
PCL_M1.7_R0.67	40	60	0.67	4.24	4.51	8.75	194.31

Table S3. Data of the measured average nanofiber diameter (including standard deviation), amount of polymer that cross the Taylor cone, and the applied flow rate at the four different values of solvent mixture A) R=4, B) R= 1,5, C) R=1 and D) R=0.66, with Z (nm), X (mg/h), and Y (mL/h).

A)

R=4	Average NFs diameter (Z)	Polymer Taylor cone (X)	Flow rate (Y)
	727 ± 323	85.18	0.5
	809 ± 296	170.36	1
	1081 ± 650	255.54	1.5
	1545 ± 850	340.72	2

B)

R=1.5	Average NFs diameter (Z)	Polymer in Taylor cone (X)	Flow rate (Y)
	900 ± 191	90.7	0.5
	1033 ± 323	181.5	1
	1568 ± 419	272	1.5
	1919 ± 512	363.1	2

C)

R=1	Average NFs diameter (Z)	Polymer Taylor cone (X)	Flow rate (Y)
	1057 ± 193	93.86	0.5
	1541 ± 330	187.72	1
	2073 ± 517	281.58	1.5
	2296 ± 748	375.44	2

D)

R=0.67	Average NFs diameter (Z)	Polymer in Taylor cone (X)	Flow rate (Y)
	1324 ± 290	97.15	0.5
	2108 ± 477	194.3	1
	2685 ± 800	291.4	1.5
	2925 ± 783	388.63	2

Table S4. Data of the average nanofiber diameter, viscosity, and the solvent R value at the four different flow rates used for the fabrication of the PCL NFs. A) 0.5 mL/h, B) 1 mL/h, C) 1.5 mL/h and D) 2 mL/h, with Z (nm), X (mg/h), and Y (mL/h).

A)

**Q=0,5 mL/h**	**Average NFs diameter (Z)**	**Viscosity (X)**	**R value (Y)**
	727 ± 322	1628	4
	900 ± 178	2572	1.5
	1057 ± 775	2834	1
	1324 ± 290	3096	0.67

B)

**Q=1 mL/h**	**Average NFs diameter (Z)**	**Viscosity (X)**	**R value (Y)**
	809 ± 295	1628	4
	1033 ± 340	2572	1.5
	1541 ± 330	2834	1
	2108 ± 479	3096	0.67

C)

**Q=1,5 mL/h**	**Average NFs diameter (Z)**	**Viscosity (X)**	**R value (Y)**
	1081 ± 650	1628	4
	1568 ± 658	2572	1.5
	2073 ± 517	2834	1
	2685 ± 800	3096	0.67

D)

**Q=2 mL/h**	**Average NFs diameter (Z)**	**Viscosity (X)**	**R value (Y)**
	1545 ± 850	1628	4
	1919 ± 785	2572	1,5
	2296 ± 748	2834	1
	2925 ± 783	3096	0.67

Table S5. Kinetic fitting parameters for Higuchi, Peppas-Sahlin and First-order models.

Release	Higuchi		Peppas-Sahlin				First Order		
	k_H	R^2	k_1	k_2	m	R^2	Q_∞	k	R^2
**Release 1**	1.150	0.227	7.872	1.639	0.154	0.970	31.71	0.025	0.558
**Release 2**	0.290	-1.122	3.227	0.992	0.086	0.973	8.08	0.073	0.759
**Release 3**	2.145	-3.327	32.58	0.008	0.109	0.799	64.27	0.096	0.991
**Release 4**	0.416	-3.093	9.634	7.69×10^{-6}	0.041	0.989	12.19	0.143	0.949
**Release 5**	1.914	-2.543	33.03	0.020	0.090	0.868	57.50	0.100	0.992
**Release 6**	0.368	-3.420	7.460	3.96×10^{-8}	0.062	0.971	10.85	0.076	0.930

k_H: Higuchi constant; k_1: Fickian diffusion coefficient; k_2: relaxation coefficient; m: diffusional exponent; $Q\infty$: maximum release (µg/mg); k: first-order rate constant (min^{-1}).

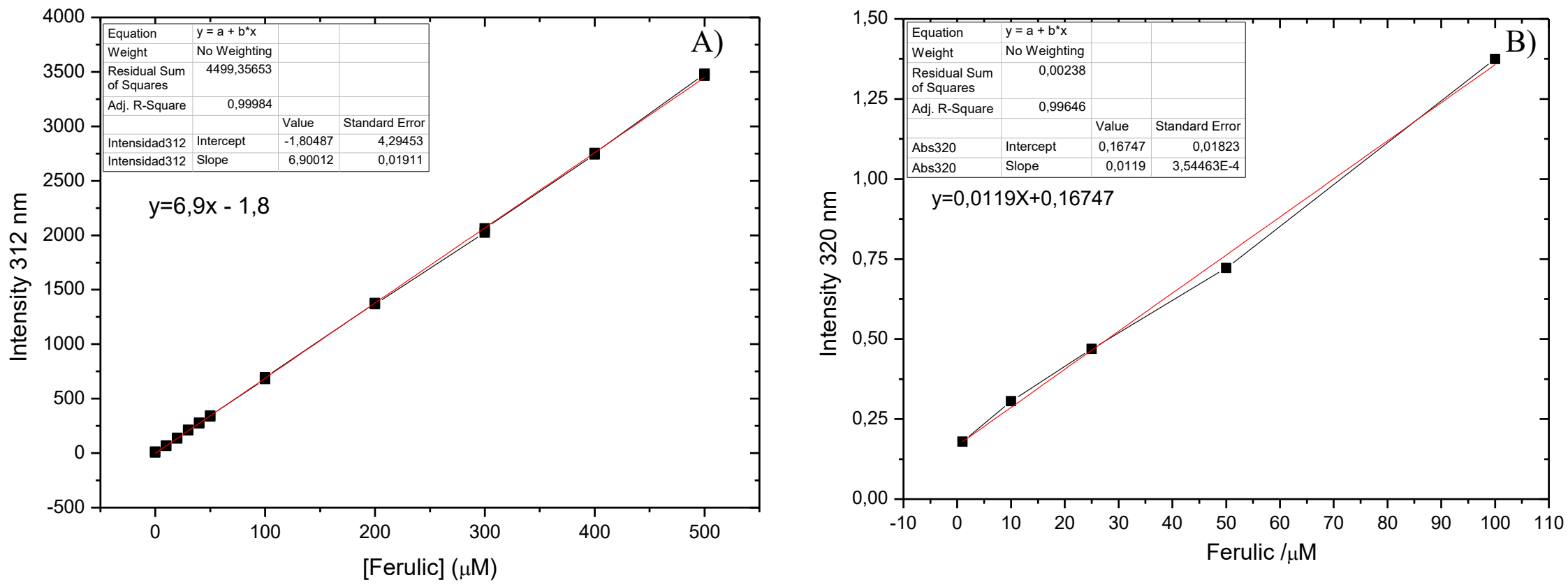

Figure S1. A) Calibration straight for ferulic acid between 1 and 500 mM in water. B) Calibration straight for ferulic acid between 1 and 100 mM in THF.

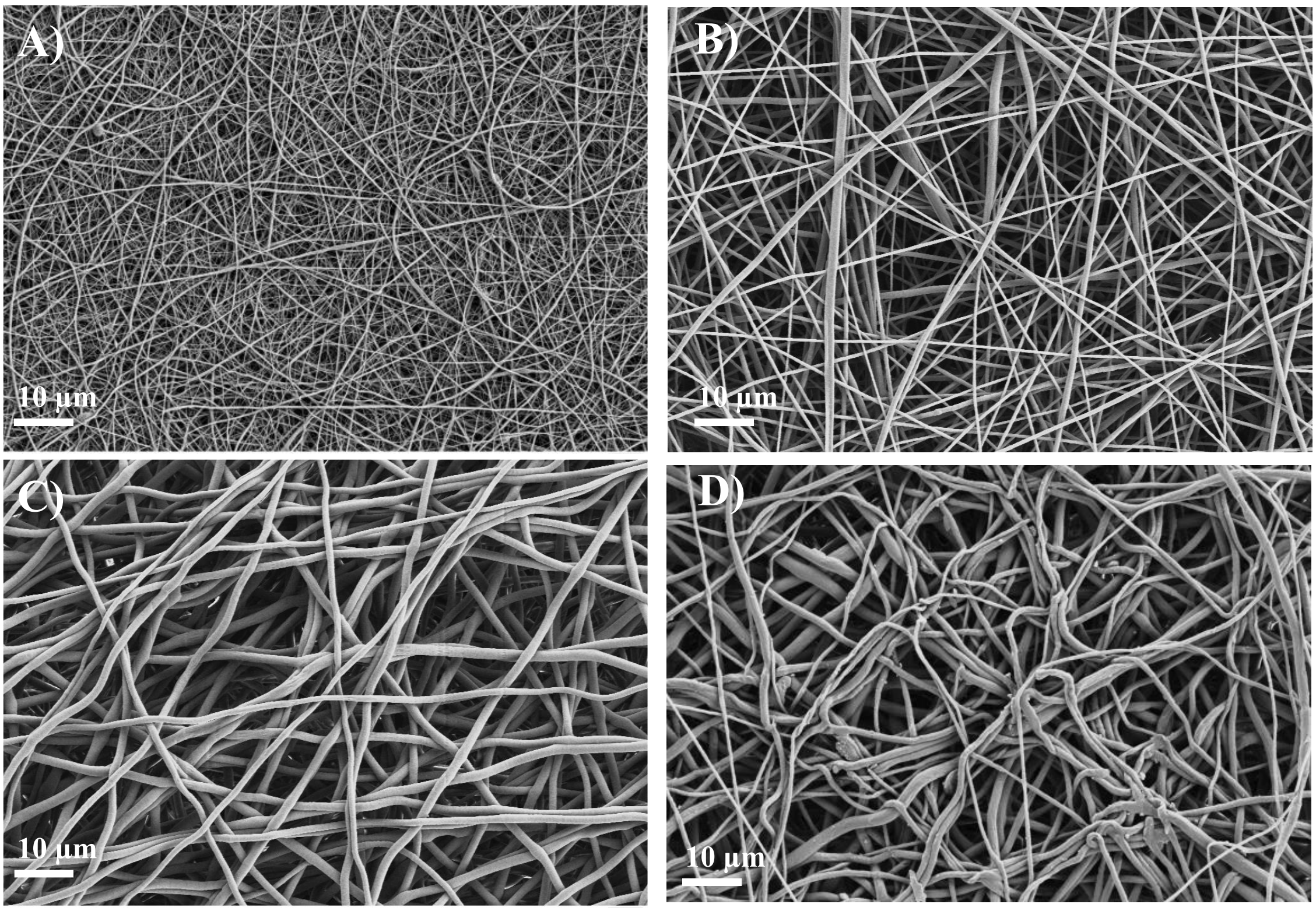

Figure S2. Representative SEM images of the PCL NFs obtained at a R value of 1.5 at different flow rates A) 0.5 mL/h, B) 1 mL/h C) 1.5 mL/h and D) 2 mL/h.

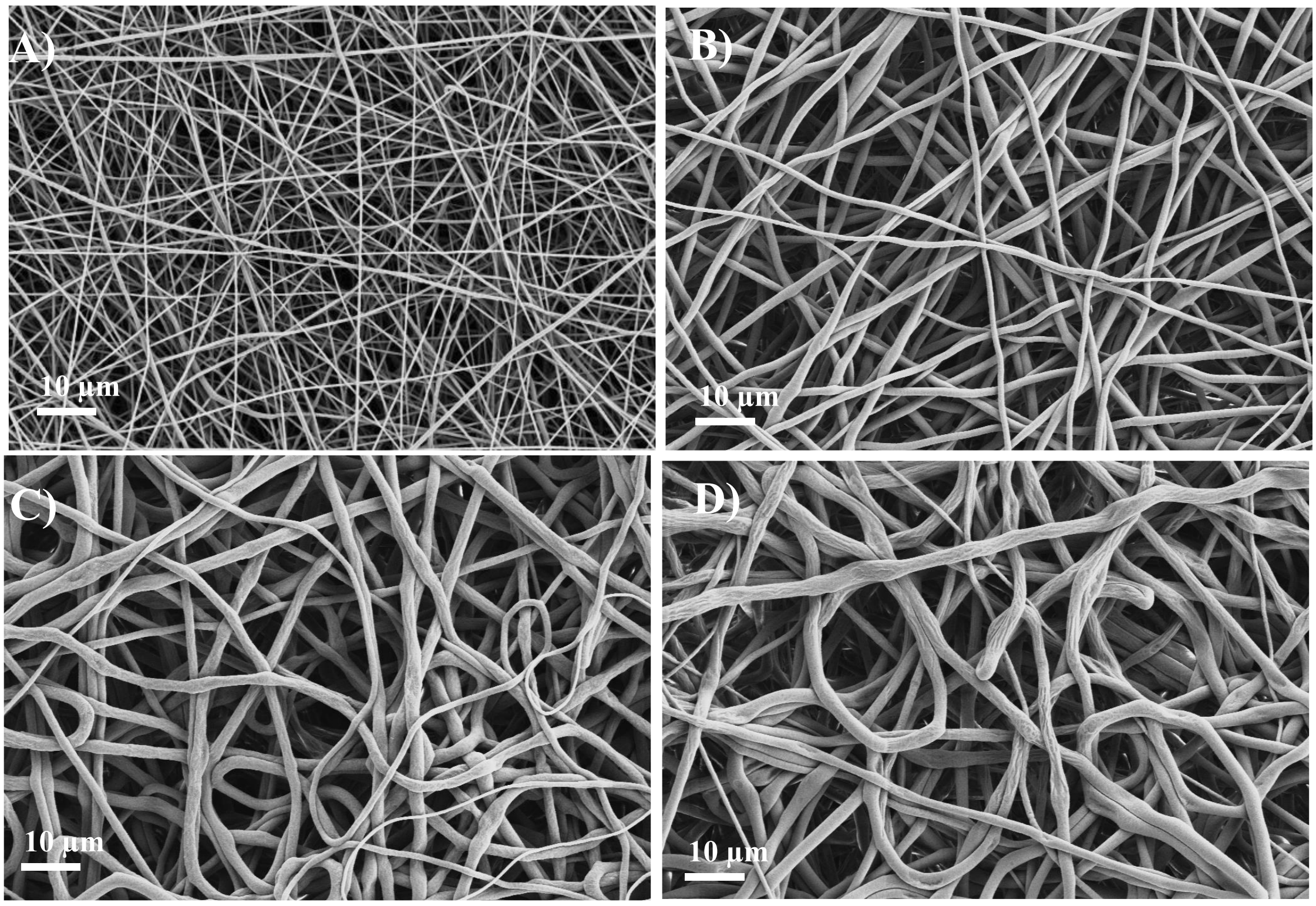

Figure S3. Representative SEM images of the PCL NFs obtained at a R value of 1 at different flow rates A) 0.5 mL/h, B) 1 mL/h C) 1.5 mL/h and D) 2 mL/h.

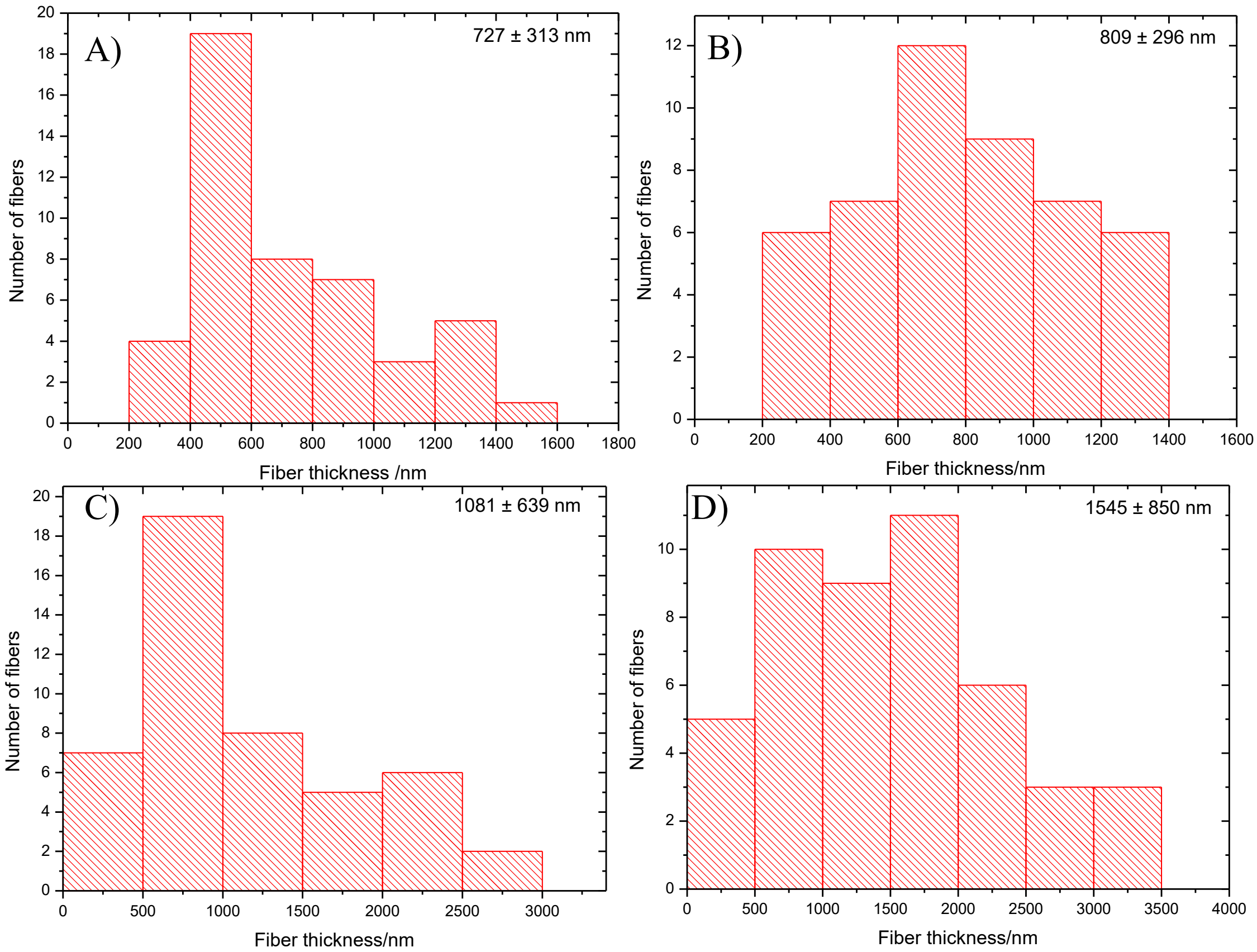

Figure S4. Nanofiber diameter distribution, including the standard deviation, for the PCL NFs obtained at a R value of 4 at different flow rates A) 0.5 mL/h, B) 1 mL/h C) 1.5 mL/h and D) 2 mL/h

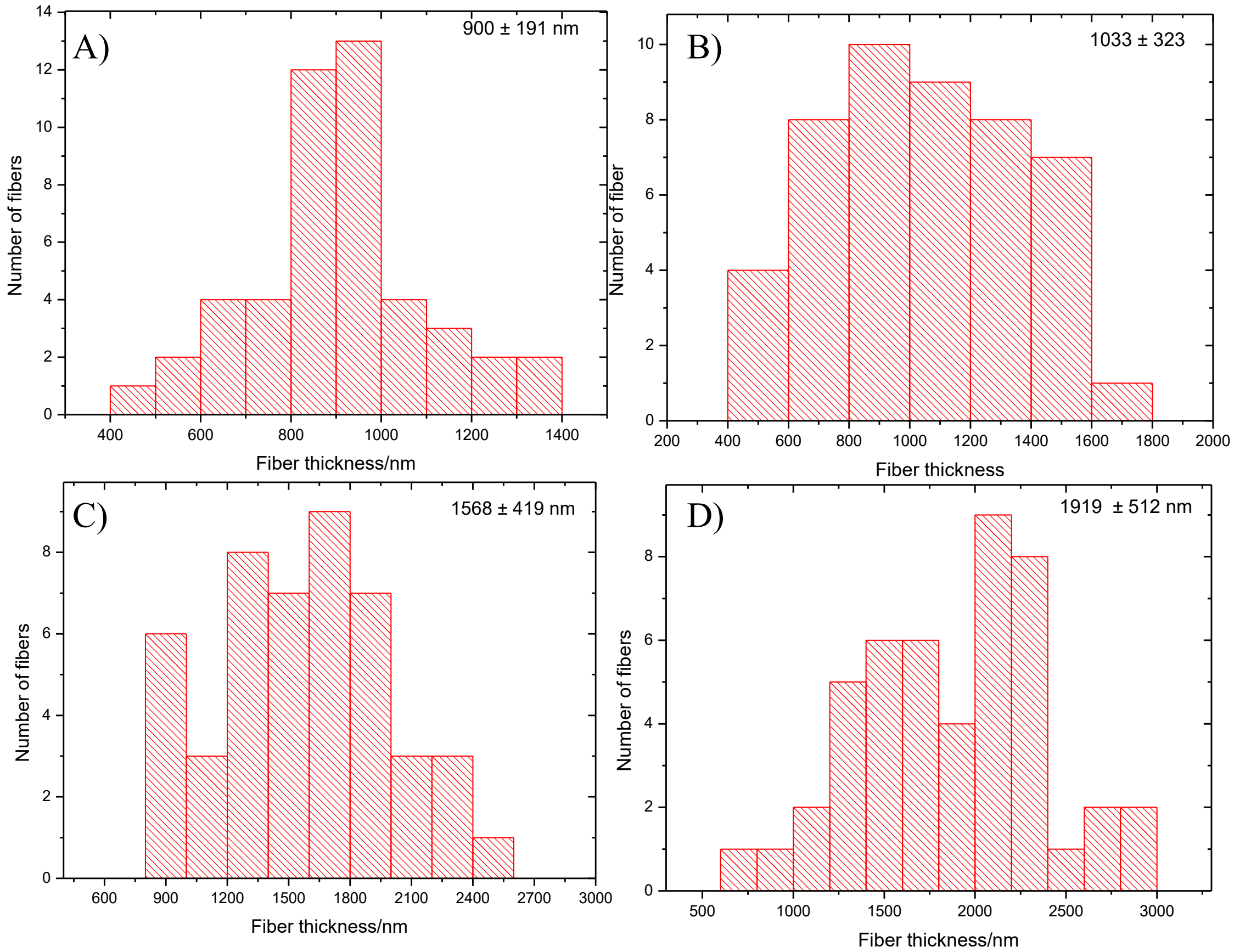

Figure S5. Nanofiber diameter distribution, including the standard deviation, for the PCL NFs obtained at a R value of 1.5 at different flow rates A) 0.5 mL/h, B) 1 mL/h C) 1.5 mL/h and D) 2 mL/h.

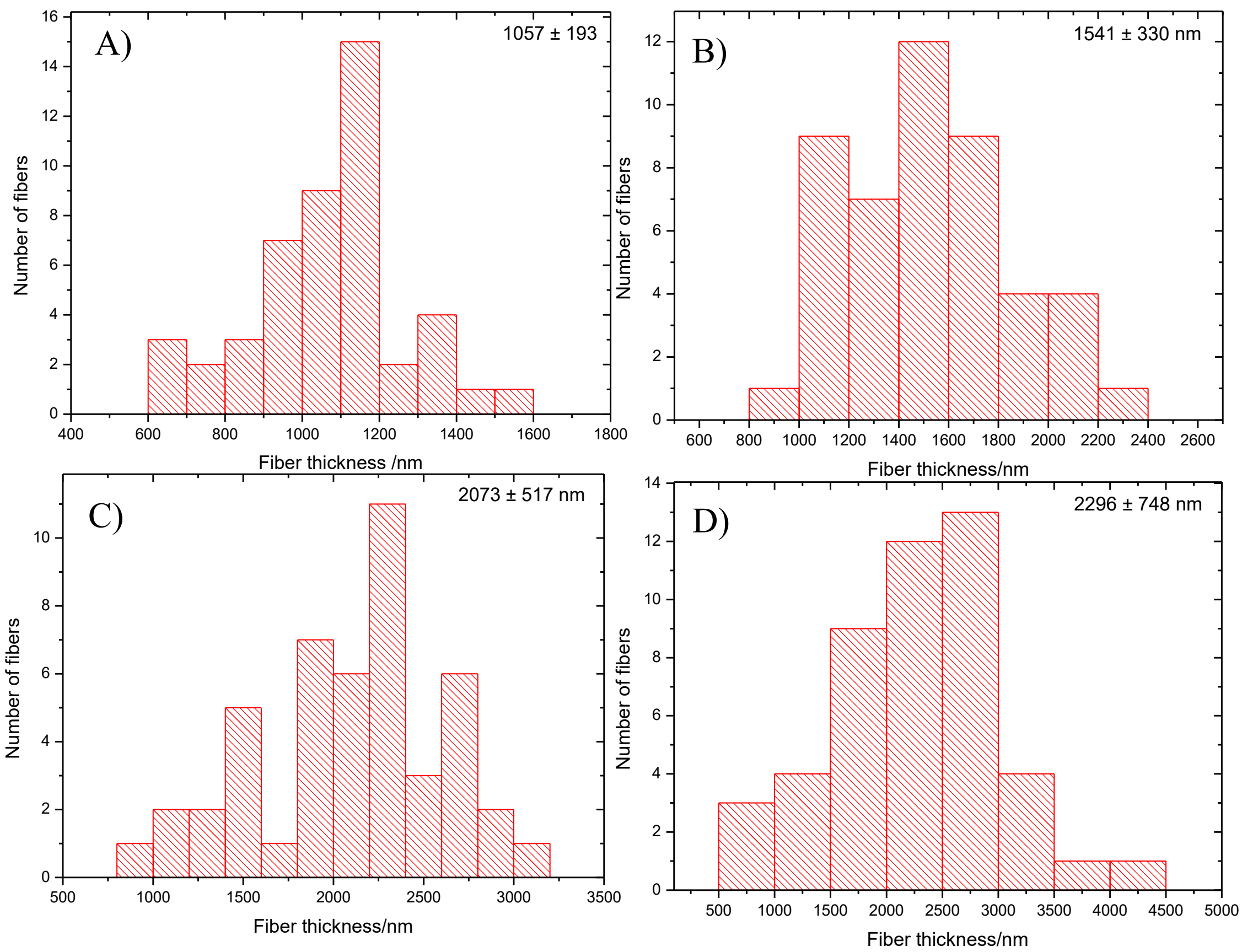

Figure S6. Nanofiber diameter distribution, including the standard deviation, for the PCL NFs obtained at a R value of 1 at different flow rates A) 0.5 mL/h, B) 1 mL/h C) 1.5 mL/h and D) 2 mL/h.

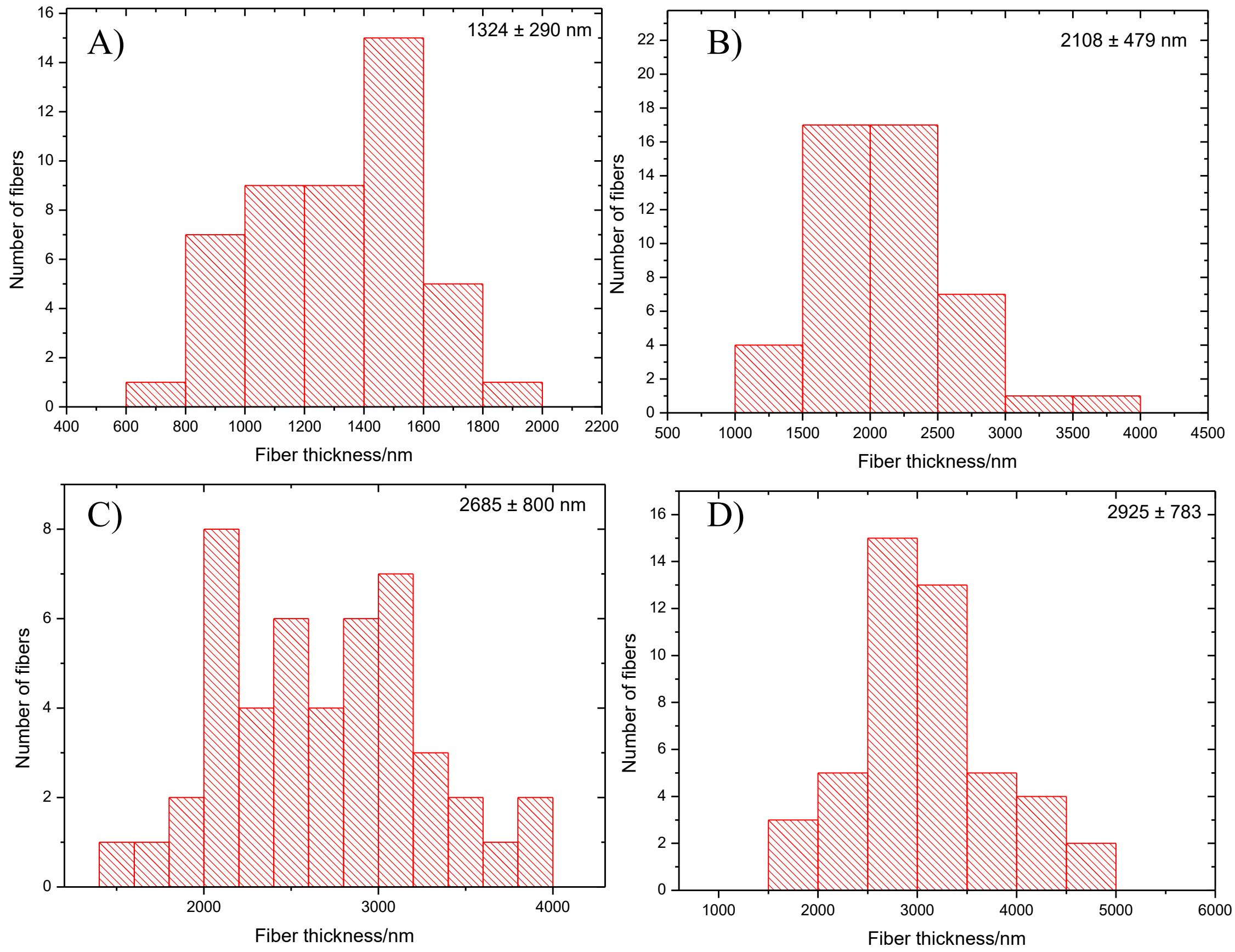

Figure S7. Nanofiber diameter distribution, including the standard deviation, for the PCL NFs obtained at a R value of 0.67 at different flow rates A) 0.5 mL/h, B) 1 mL/h C) 1.5 mL/h and D) 2 mL/h.

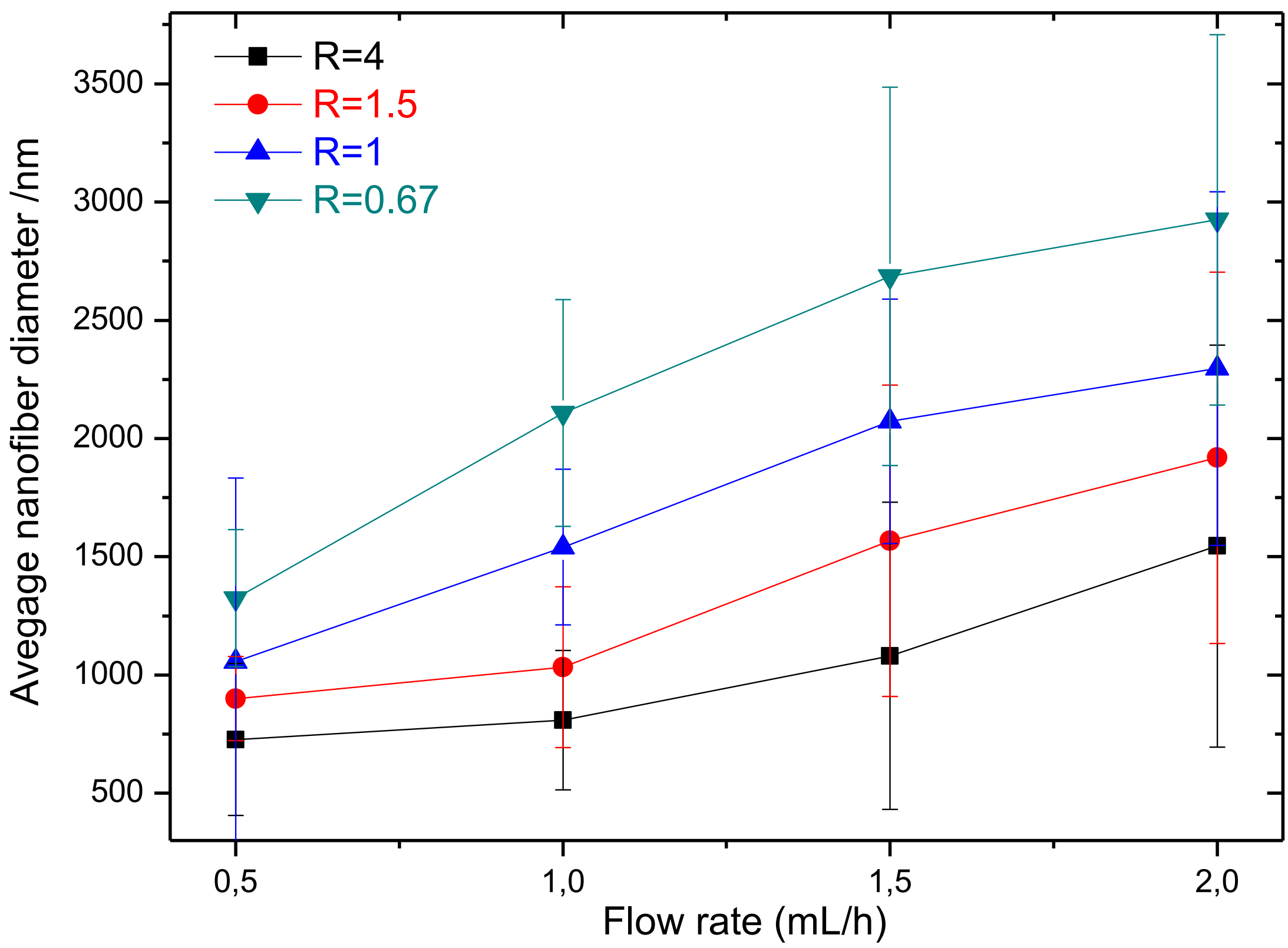

Figure S8. Average nanofiber diameter (including the standard deviation) for PCL NFs in function of the flow rate value at the four solvent ratios investigated. R=4 (black line), R=1.5 (red line), R=1 (blue line) and R=0.67 (green line).

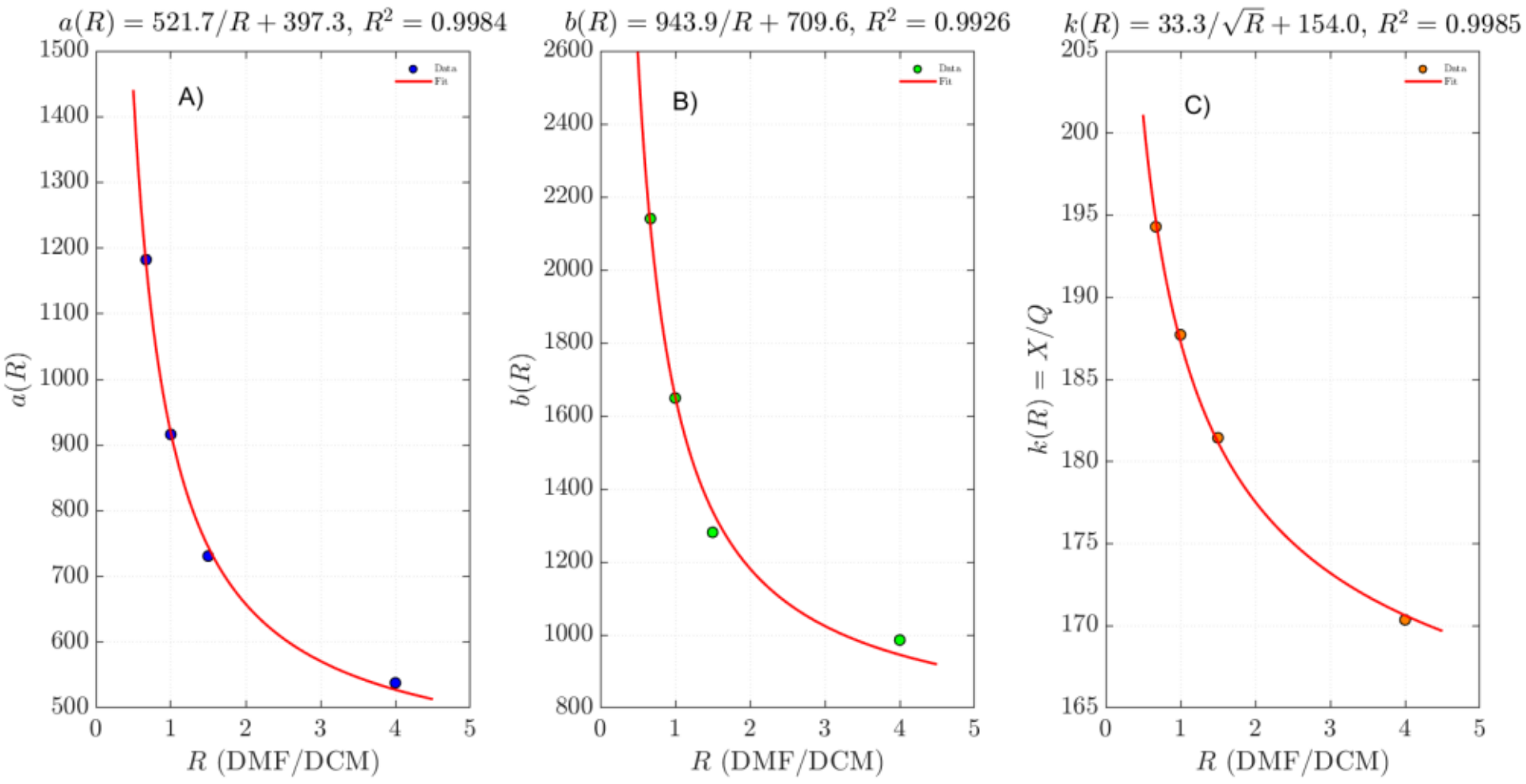

Figure S9. Dependence of the fitted parameters of the response-surface model on the solvent ratio R. A) $a(R) = 521.7/R + 397.3$. B) $b(R) = 943.9/R + 709.6$. C) $k(R) = 33.3/\sqrt{R} + 154.0$, which relates the amount of polymer in the Taylor cone X and the flow rate Q through $X = k(R) \cdot Q$. Symbols are experimental values and solid red lines are the corresponding inverse or inverse-square-root fits.

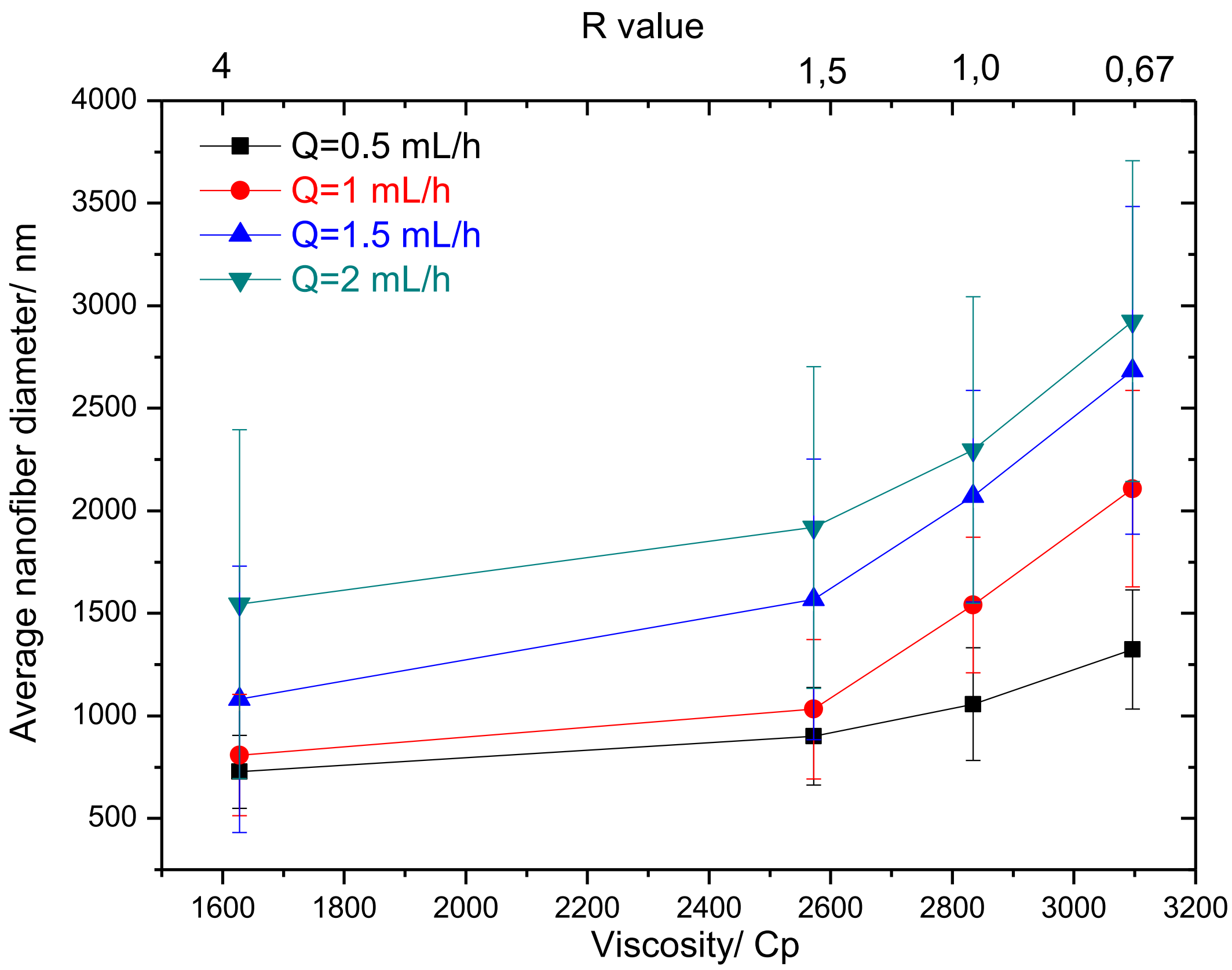

Figure S10. Average nanofiber diameter (including the standard deviation) for PCL NFs in function of the R value for the four flows rate investigated. Q=0.5 mL/h (black line), Q=1 mL/h (red line), Q=1.5 mL/h (blue line) and Q=2 mL/h (green line).

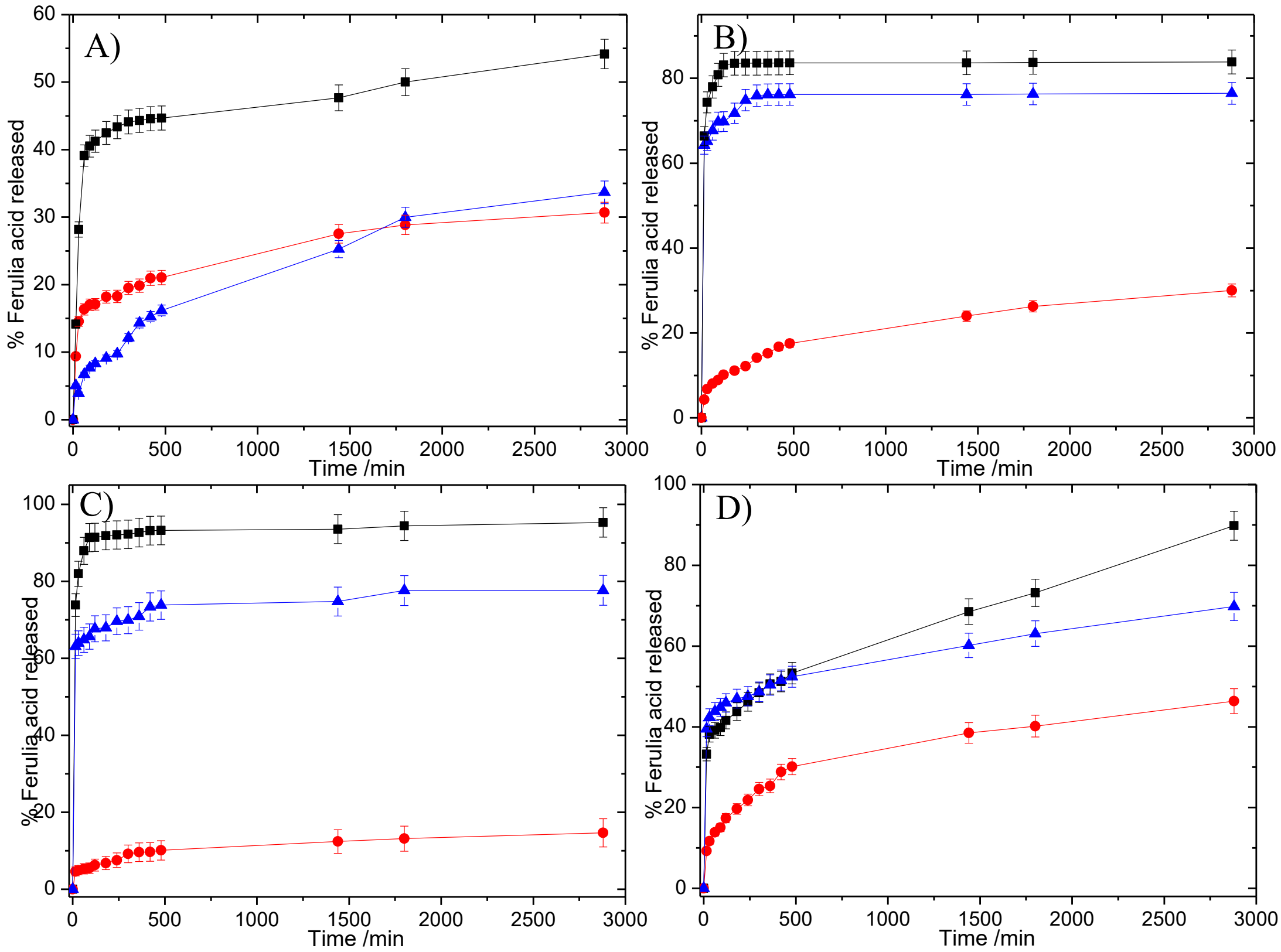

Figure S11. Percentage of FA released in function of time for PCL NFs prepared with 6% (black square), 2% (blue triangles) and 0.5% (red circle) of FA and for A) R=6 and Q=1.5 mL/h, B) R=0.67 and Q=0.5 mL/h B) R=4 and Q=1.5 mL/h and C) R=4 and Q=0.5 mL/h

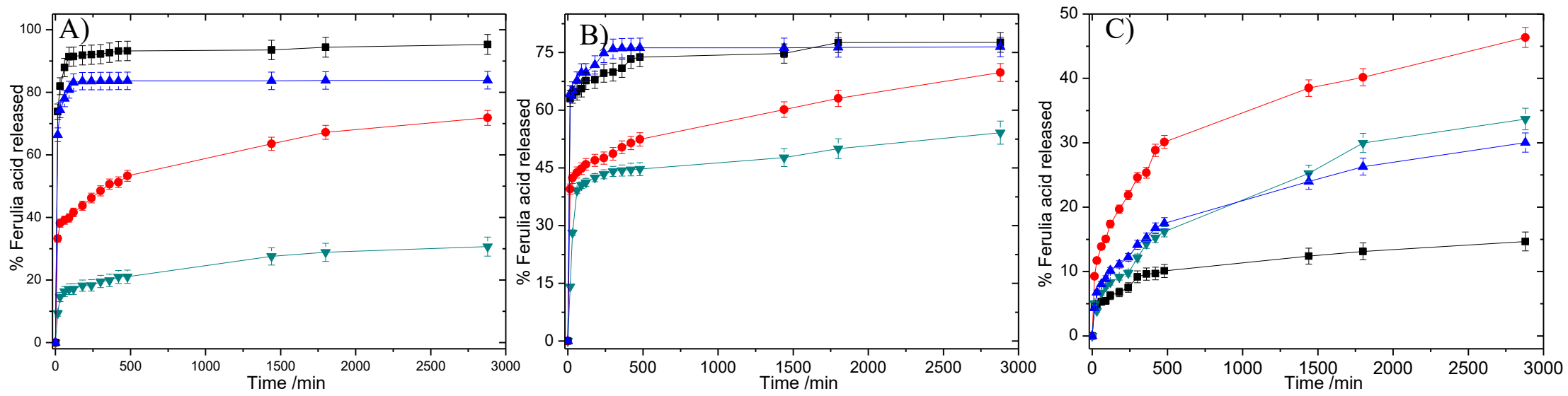

Figure S12. Percentage of ferulic acid released in function of time for PCL_R4_Q1.5 (black square), PCL_R4_Q0.5 (blue triangles), PCL_R0.67_Q1.5 (green triangles) and PCL_R0.67_Q0.5 (blue triangles) with A) 6% of ferulic acid B) 2% of ferulic acid and C) 0.5% of FA.
